# Global Diversity, Host Associations, and New Insights into Aigialaceae, Astrosphaeriellaceae, and Pseudoastrosphaeriellaceae

**DOI:** 10.3390/jof11120834

**Published:** 2025-11-25

**Authors:** Danushka S. Tennakoon, Nimali I. de Silva, Ning Xie, Sinang Hongsanan

**Affiliations:** Shenzhen Key Laboratory of Microbial Genetic Engineering, College of Life Science and Oceanography, Shenzhen University, Shenzhen 518060, China; danushkasandaruwanatm@gmail.com (D.S.T.); nimalindeewari@gmail.com (N.I.d.S.); ning.xie@szu.edu.cn (N.X.)

**Keywords:** check list, multi-gene phylogeny, new species, new host records, saprobes

## Abstract

During a survey of plant litter-associated microfungi in Guangdong and Jiangxi Provinces, China, several specimens that have carbonaceous ascomata were collected. Morphological characteristics combined with multi-gene (LSU, SSU, and *tef1-α*) phylogeny revealed that they belong to the Aigialaceae, Astrosphaeriellaceae, and Pseudoastrosphaeriellaceae families. Phylogenetic analyses were conducted using Maximum Likelihood (ML) and Bayesian Inference (BI) approaches. *Caryospora pruni* and *Pseudoastrosphaeriella zingiberacearum* are introduced as new species, and *Astrosphaeriella bambusae*, *C. quercus*, *Fissuroma caryotae*, and *Neoastrosphaeriella aquatica* are introduced as new host records. In addition, *Caryospora minima* is synonymized under *C. aquatica* based on close morphological and phylogenetic relationships. All the newly introduced species fit well with their respective generic concepts and can be distinguished from closely related species in their morphology and DNA molecular data. The new host records also provide similar morphological characteristics to their respective type species, and multi-gene phylogeny analyses also offer evidence for their placements. In addition, we compiled the geographical distribution and host associations of species in Aigialaceae, Astrosphaeriellaceae, and Pseudoastrosphaeriellaceae. This provides a database for future studies to understand the ecological interactions and geographical variations.

## 1. Introduction

**Figure 1:**
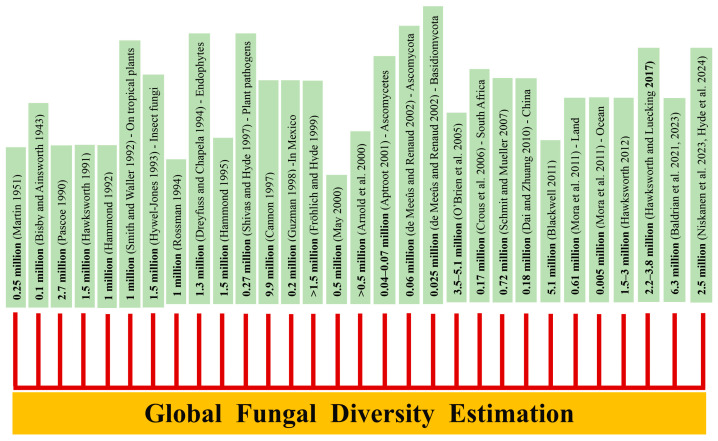
Estimations of the global number of fungal species [19,20,21,22,23,24,25,26,27,28,29,30,31,32,33].

Global fungal diversity is a critical aspect of global biodiversity, and realistic estimations have been controversial for decades (Figure 1). Mycologists have used various estimation criteria to achieve the realistic fungal diversity over time [1,2,3,4,5,6,7,8]. For instance, based on the plant-to-fungi ratio of 1:6, Hawksworth [1] estimated that there are approximately 1.5 million species. This assessment was revised by Hammond [2] and Rossman [3] and assessed to have 1 million species. Subsequently, the number of fungal species worldwide fluctuated significantly with varying numbers, such as 1.5 million [9], 9.9 million [10], 0.5 million [4], >1.5 million [11], 0.5–9.9 million [12], 3.5–5.1 million [13], 0.72 million [14], 5.1 million [5], and 1.5–3 million [15]. In 2017, Hawksworth and Lücking adjusted the fungal diversity figure to 2.2–3.8 million species, considering plant-to-fungi ratios, hidden cryptic species, high-throughput data, unexplored habitats, and the ongoing discovery of novel taxa. This was further expanded to 11.7–13.2 million [7] and to 6.3 million species [16,17] with the consideration of high-throughput sequencing (HTS)-powered metabarcoding approaches.

This dramatic increase in estimated diversity is attributed to advancements in DNA sequencing technologies and the comprehensive studies that collectively assess fungal diversity across various ecosystems. However, despite this vast diversity of fungal communities, only about 150,000 species have been formally described, highlighting a considerable gap in our understanding of fungal life forms and their ecological roles [18]. The discrepancy between estimated and described species numbers emphasizes the need for extensive investigations into fungal diversity, especially within unexplored niches and biodiversity hotspots, substrates, and regions.

China is recognized as a global hotspot for fungal diversity, particularly due to the vast geographical and ecological variety (e.g., forests, grasslands, lakes, mangroves, mountains, rivers, and seas), which contributes to a rich array of fungal species [34,35,36]. In particular, the highly variable climate and luxuriant vegetation in China make favorable settings for the growth and reproduction of fungal species [37,38]. Over the past three decades, mycologists in China have significantly contributed to the inclusive range of fungal discoveries [34,38,39,40,41,42,43,44,45]. Initially, fungal taxonomy studies in China were mainly based on morphological characteristics coupled with hand-drawn illustrations with descriptions and taxonomic keys [46,47,48,49]. However, this has undergone a revolutionary shift from traditional morphology-based identification to molecular-based techniques with the rapid evolution of DNA sequencing technology [45,50,51,52]. In addition, current HTS platforms generate millions of barcode reads within hours, enabling researchers to resolve cryptic species, detect rare or dark taxa in complex substrates, and reconstruct phylogenies [53,54,55]. As a result, China has emerged as a key contributor to global fungal research, with an increasing number of studies and novel discoveries. Currently, China accounts for almost 7% of recorded fungal species, occupying the second-highest position worldwide [38].

Therefore, our research group at the College of Life Science and Oceanography in Shenzhen University has been conducting investigations into the microfungal diversity of plant litter substrates (e.g., dead wood, leaves, branches, seeds, twigs, and fruits) in China. Mainly, we are focusing on their geographical distribution, host associations, morphological characteristics, and phylogenetic affinities [56,57]. As a result, we collected six plant litter specimens bearing carbonaceous ascomata from *Arenga undulatifolia*, *Citrus maxima*, *Hedychium coronarium*, *Metroxylon sagu*, *Phoenix paludosa*, and *Prunus persica*. They apparently belong to the families Aigialaceae, Astrosphaeriellaceae, and Pseudoastrosphaeriellaceae based on ascomata characteristics (e.g., carbonaceous, conical to hemispherical shape) and the previous literature [58,59,60]. The objectives of this study are (1) to identify the isolated taxa using both morphology and phylogenetic approaches and (2) to provide an updated checklist (host occurrence and global distribution) of the species in Aigialaceae, Astrosphaeriellaceae, and Pseudoastrosphaeriellaceae. In particular, this study offers a database for future studies to understand the worldwide distribution and host connections of those families.

## 2. Materials and Methods

### 2.1. Samples Collection, Microscopic Observations, and Fungal Isolation

Decaying plant litter samples (dead stems and pericarp of fruit) were collected from Guangdong and Jiangxi Provinces during 2024–2025. Collected specimens were taken back to the laboratory in sealed envelopes for morphology examination. All the specimens were incubated in a plastic box with a wet tissue paper (two days). Initially, the macro-morphological characteristics were checked using a stereomicroscope (AXIOSKOP 2 PLUS Series, Göttingen, Germany), and mainly surface characteristics of ascomata, such as color, shape, and position of the host surface (e.g., immersed, semi-immersed, and erumpent or superficial), were recorded. Ascomata were manually sectioned using a razor blade and mounted in water on a slide to observe their microscopic features (e.g., shape, the arrangement of peridium cells). Microscopic slides were prepared using the needle of an aseptic syringe and distilled water. Micro-morphological characteristics were examined using a Nikon ECLIPSE Ni-U compound microscope (Nikon Corporation, Tokyo, Japan), equipped with a Canon Axiocam 506 color digital camera (Hanover, Germany). Indian ink was used to determine the presence of the mucilaginous sheath of ascospores. Lactoglycerol and nail polish were used to prepare the permanent slides for future studies. Measurements were obtained using NIS-Elements version 5.10 imaging software when capturing photographs. Photo plates were prepared using Adobe Photoshop CS6 Extended version 10.0 software (Adobe Systems, San Jose, CA, USA).

Single ascospore isolation was conducted, and germinated spores were processed by following the methods described in Senanayake et al. [61]. Pure cultures were grown in potato dextrose agar (PDA) medium at room temperature (24–28 °C). Culture characteristics, such as color, density, shape, elevation, surface, edge, consistency, and margin, were observed after three weeks. Type specimens were deposited at room temperature (24–28 °C) in the Herbarium of Fungi, Shenzhen University, Shenzhen, China. Living cultures were deposited in the Culture Collection of Microbial Shenzhen University (MBSZU), Shenzhen University, Shenzhen, China. The new taxa were registered in Index Fungorum numbers (https://www.indexfungorum.org/names/Names.asp, accessed on 20 September 2025).

### 2.2. DNA Extraction, PCR Amplification, and Sequencing

The scraped fungal mycelia (grown on PDA for 4 weeks at 24–28 °C) were used for the DNA extraction (for *Astrosphaeriella bambusae*, *Caryospora pruni*, and *Pseudoastrosphaeriella zingiberacearum*). Initially, axenic mycelium (50–100 mg) was crushed to a fine powder using glass beads (acid-washed, 212–300 µm, Norcross, GA 30071, USA). The extraction process was conducted using a DNA extraction kit (E.Z.N.A. Tissue DNA Kit, D3396-02, Omega Biotek, Inc., Norcross, GA 30071, USA) following the manufacturer’s protocol with various chemical solutions such as BL Buffer, HBC Buffer, DNA Wash Buffer, Elution Buffer, and OB Protease Solution. Some fungal species did not germinate (*Caryospora quercus*, *Fissuroma caryotae*, and *Neoastrosphaeriella aquatica* species), and thus, their DNA extraction was performed using fruiting bodies. The E.Z.N.A. Forensic DNA Kit-D3591-01 (Omega Biotek, Inc., Norcross, GA 30071, USA) was used for their DNA extraction. Polymerase chain reactions (PCR) were conducted to amplify regions of the small nuclear ribosomal subunit rDNA (SSU), large nuclear ribosomal subunit rDNA (LSU), and translation elongation factor 1-alpha gene (*tef1-α*) genes, using the primer pairs as mentioned in Table 1. The total volume of the PCR reaction was 25 µL containing 12.5 µL 2X PCR MasterMix (TIANGEN Co., Beijing, China), 9.5 µL double-distilled water, 1 µL genomic DNA, and 1 µL of each forward and reverse primer (stock of 10 pM). Thermal cycle programs for LSU, SSU, and *tef1-α* genes were followed as mentioned in Tennakoon et al. [56]. PCR products were detected by NanoDrop One (Thermo Fisher Scientific, Waltham, MA, USA) and 1% agarose gels, and then sequenced by Beijing Liuhe BGI Genomics Co., Ltd., Beijing, China, using the same primers used for PCR amplification. The generated sequences were deposited in GenBank, and accession numbers were obtained (Table 2).

**Table 1:** Forward and reverse primer information of LSU, SSU, and *tef1-α* gene regions.

Locus	Primers	Reference
LSU	Forward: LR0R GTACCCGCTGAACTTAAGC	[62]
	Reverse: LR5 ATCCTGAGGGAAACTTC	
SSU	Forward: NS1 GTAGTCATATGCTTGTCTC	[63]
	Reverse: NS4 CTTCCGTCAATTCCTTTAAG	
*tef1-α*	Forward: EF1-983F GCYCCYGGHCAYCGTGAYTTYAT	[64]
	Reverse: EF1-2218R ATGACACCRACRGCRACRGTYTG	

### 2.3. Phylogenetic Analyses

Based on the corresponding Sanger sequencing chromatograms, sequences were initially checked with BioEdit v 7.0.5.3 [65]. Ambiguous regions from the ends of raw sequencing fragments were manually trimmed and assembled into consensus sequences using SeqMan Pro Version 7.1.0 (DNASTAR, Inc., Madison, WI, USA). Consensus sequences were used for BLAST searches in GenBank (https://blast.ncbi.nlm.nih.gov/Blast.cgi). The BLAST search results and sequences from the latest publications were used to obtain sequence data for the phylogenetic analyses [59,60,66]. Individual gene regions were aligned using MAFFT v.7 (http://mafft.cbrc.jp/alignment/server/, accessed on 1 September 2025) and trimmed to remove ambiguous bases using TrimAl 1.2 [67,68]. Aligned sequences were combined using BioEdit v.7.2.5 [65].

Two phylogenetic analyses were conducted, such as Maximum Likelihood (ML) and Bayesian Inference (BI). Maximum Likelihood analysis was performed via the online portal CIPRES Science Gateway v. 3.3 [69], with RAxML-HPC v.8 on XSEDE (8.2.12) tool, using default settings but following the adjustments: the GTRGAMMA nucleotide substitution model and 1000 rapid bootstrap replicates. Bayesian inference analysis was performed using MrBayes 3.2.1 [70]. Six simultaneous Markov chains were run for 3,000,000 generations, with trees sampled every 100th generation (GTR + I + G substitution model), until it was stopped when the standard deviation of split frequencies between the two simultaneous runs dropped below 0.01. The first 20% of generated trees were discarded as burn-in, and the rest (80%) were used to calculate the posterior probability. The evolutionary model of nucleotide substitution for both ML and BI analyses was selected independently for each locus using MrModeltest 2.3 [71]. Phylogenetic trees were visualized with FigTree v.1.4.0 [72]. Trees were edited using Microsoft PowerPoint (Microsoft, 2010) and Adobe Illustrator^®^ CS6 v.26.0 (Adobe Systems, San Jose, CA, USA). The newly generated sequences were submitted to GenBank (https://ncbi.nlm.nih.gov, accessed on 10 September 2025). The final alignment was submitted to TreeBASE, submission ID:32279 (http://www.treebase.org/, accessed on 10 September 2025).

### 2.4. Geographical Distribution and Host Associations

In this study, we gathered and documented the geographical distribution and host occurrence data of 165 species from the families Aigialaceae, Astrosphaeriellaceae, and Pseudoastrosphaeriellaceae. The data contained 29 species in Aigialaceae, which are included within five genera (*Aigialus*, *Fissuroma*, *Neoastrosphaeriella*, *Posidoniomyces*, and *Rimora*); 126 species in Astrosphaeriellaceae, which are included within 10 genera (*Aquatospora*, *Astrosphaeriella*, *Astrosphaeriellopsis*, *Caryospora*, *Javaria*, *Mycopepon*, *Pithomyces*, *Pteridiospora*, *Quercicola*, and *Xenoastrosphaeriella*); and 10 species in Pseudoastrosphaeriellaceae, which are included within three genera (*Carinispora*, *Pseudoastrosphaeriella*, and *Pseudoastrosphaeriellopsis*). The required data were sourced from Species Fungorum (https://www.speciesfungorum.org, accessed on 20 September 2025), MycoBank (https://www.mycobank.org/), reputable journal publications, authoritative books, the U.S. National Fungus Collections Fungus-Host Database [73], and the Catalogue of Life Checklist (https://www.catalogueoflife.org/, accessed on 20 September 2025). All the collected data were listed with appropriate references (Table 3). In total, 154 publications were reviewed to compile data for 164 species, accessed via Google Scholar and Research Gate. The nomenclatural validity of the taxa was confirmed using MycoBank (https://www.mycobank.org/, accessed on 25 September 2025). We included only taxa that were validly published, morphologically described, and assigned to one of the above families with clearly indicated host and/or locality data. Records with ambiguous identifications, unverified host information, or duplicate entries were excluded. Publications and records from 1800 to 2025 were considered. However, we noted that many species remain unverified by molecular data, and their identification and phylogenetic affinities require clarification through combined morphological and multi-gene phylogenetic analyses. Consequently, the distribution patterns may show slight variations. The online tool SankeyMATIC by Steve Bogart (www.sankeymatic.com, accessed on 27 September 2025) was used to visualize the species distribution through plant host families. The MapChart program (https://www.mapchart.net/index.html, accessed on 30 September 2025) was used to construct the distribution map.

**Table 2:** GenBank and culture collection accession numbers of species included in the phylogenetic study. The newly generated sequences are shown in bold. Unavailable sequences are indicated as “-”.

Taxon	Strain/Voucher Number	GenBank Accession Numbers			Reference
		LSU	SSU	*tef1-α*	
*Aigialus grandis*	MFLUCC 15-1281	MN420684	MN420694	-	[74]
*A. parvus*	BCC 32558	GU479779	GU479743	GU479843	[75]
*Astrosphaeriella bakeriana*	CBS 115556	GU301801	-	GU349015	[76]
*As. bambusae*	MFLUCC 13-0230	KT955461	-	KT955424	[58]
***As. bambusae***	**MBSZU 25-061**	**PX225037**	**PX225048**	**PX226738**	**this study**
*As. fusispora*	MFLUCC 10-0555	KT955462	KT955443	KT955425	[58]
*As. lophiostomopsis*	HKUCC 2984	GU205215	GU205232	-	[77]
*As. neofusispora*	MFLUCC 11-0161	KT955463	KT955444	KT955426	[58]
*As. neostellata*	CX003	MN629351	MN629353	MN635787	[78]
*As. neostellata*	MFLUCC 11-0625	KT955464	-	-	[58]
*As. stellata*	MFLUCC10-0555	JN846723	JN846733	-	[79]
*As. thailandica*	MFLUCC 11-0191	KT955465	KT955445	KT955427	[58]
*As. thysanolaenae*	MFLUCC 11-0186	KT955466	KT955446	KT955428	[58]
*Astrosphaeriellopsis caryotae*	MFLUCC 13-0830	MF588990	MF588980	-	[59]
*Carinispora nypae*	BCC 36316	-	GU479749	GU479849	[75]
*C. aquatica*	MFLUCC 11-0008	MH057847	MH057850	-	[80]
*C. aquatica*	HKAS 112608	OP377899	OP377986	-	[81]
*C. aquatica*	MFLUCC 18-1030	MT627666	MT864295	MT954394	[60]
*C. minima*	taxon:492516	EU196550	EU196551	-	[82]
***C. pruni***	**MBSZU 25-055**	**PX225040**	**PX225051**	**PX226741**	**this study**
***C. pruni***	**MBSZU 25-056**	**PX225041**	**PX225052**	**PX226742**	**this study**
***C. pruni***	**MBSZU 25-057**	**PX225042**	**PX225053**	**PX226743**	**this study**
*C. quercus*	MFLU 18-2151	MK347979	MK347869	-	[83]
*C. quercus*	MFLUCC 17-2342	MN913681	MT864311	-	[60]
*C. quercus*	MFLUCC 17-2323	MN913683	MT864309	-	[60]
***C. quercus***	**SZU25-042**	**PX225043**	**PX225054**	**-**	**this study**
***C. quercus***	**SZU25-043**	**PX225044**	**PX225055**	**-**	**this study**
*C. submersa*	MFLUCC 18-1283	NG_073802	MN913720	-	[60]
*C. submersa*	MFLUCC 18-1409	MN913719	-	-	[60]
*Delitschia chaetomioides*	SMH 3253.2	GU390656	-	GU327753	[84]
*D. winteri*	AFTOL-ID 1599	DQ678077	DQ678026	DQ677922	[85]
*Fissuroma calami*	MFLUCC 13-0836	MF588993	MF588983	MF588975	[59]
*F. caryotae*	MFLU 17-1253	MF588996	MF588986	MF588979	[59]
*F. caryotae*	SNT12	MN712335	MN699322	MN744228	[86]
***F. caryotae***	**SZU25-040**	**PX225038**	**PX225049**	**PX226739**	**this study**
*F. maculans*	MFLUCC 10-0886	JN846724	JN846734	-	[79]
*F. neoaggregatum*	MFLUCC 10-0554	KT955470	KT955450	KT955432	[58]
*F. palmae*	MFLU 19-0820	MN712336	-	MN744229	[86]
*F. taiwanense*	FU30861	MG189605	MG189607	MG252072	[87]
*F. taiwanense*	FU30862	MG189606	MG189608	MG252073	[87]
*F. thailandicum*	MFLUCC 11-0189	KT955472	KT955452	KT955434	[58]
*F. wallichiae*	MFLUCC 15-0315	MN726235	MN726247	MN953045	[88]
*Neoastrosphaeriella aquatica*	MFLUCC 18-0209	MK138829	MK138789	MK132866	[89]
*N. aquatica*	SNT240	MN712338	-	MN744231	[86]
*N. aquatica*	SNT190	MN712337	MN699323	MN744230	[86]
***N. aquatica***	**SZU25-047**	**PX225039**	**PX225050**	**PX226740**	**this study**
*N. krabiensis*	MFLUCC 11-0025	JN846729	JN846739	-	[79]
*N. krabiensis*	MFLUCC 11-0022	JN846727	JN846735	-	[79]
*N. phoenicis*	MFLUCC 18-1477	MN712339	MN699324	MN744232	[86]
*N. sribooniensis*	MFLUCC 13-0834	MF588997	MF588987	MF588977	[59]
*Pithomyces caryotae*	MFLUCC 13-0828	MF588999	MF588989	MF588978	[59]
*P. licualae*	MFLUCC 17-2031	MF588995	MF588985		[59]
*Pseudoastrosphaeriella africana*	MFLUCC 11-0176	KT955474	KT955454	KT955436	[58]
*P. aquatica*	MFLUCC 18-0984	MN913742	MT864336	MT954400	[60]
*P. aquatica*	MFLUCC 18-0991	MN325076	-	MT954401	[89]
*P. aquatica*	KUMCC 19-0096	MN913746	MT627731	-	[60]
*P. bambusae*	MFLUCC 10-0885	MN913695	-	-	[60]
*P. bambusae*	KUMCC 19-0091	MN913698	-	MT954365	[60]
*P. bambusae*	KUMCC 19-0093	MN913699	MT864301	MT954363	[60]
*P. bambusae*	KUMCC 19-0095	MN913700	-	MT954364	[60]
*P. bambusae*	MFLUCC 11-0205	KT955475	KT955455	KT955437	[58]
*P. longicolla*	MFLUCC 11-0171	KT955476	-	KT955438	[58]
*P. thailandensis*	MFLUCC 14-0038	KT955479	KT955458	KT955441	[58]
*P. thailandensis*	MFLUCC 11-0144	KT955478	KT955457	KT955440	[58]
*P. thailandensis*	MFLUCC 10-0553	KT955477	KT955456	KT955439	[58]
***P. zingiberacearum***	**MBSZU 25-058**	**PX225045**	**PX225056**	**PX226744**	**this study**
***P. zingiberacearum***	**MBSZU 25-059**	**PX225046**	**PX225057**	**PX226745**	**this study**
***P. zingiberacearum***	**MBSZU 25-060**	**PX225047**	**PX225058**	**PX226746**	**this study**
*Pseudoastrosphaeriellopsis kaveriana*	PUFD33	MG947595	MG947598	MG968955	[58]
*Pteridiospora bambusae*	MFLU 10-0071	MG831565	MG831566	MG833012	[90]
*Pt. chiangraiensis*	MFLUCC 11-0162	KT955480	KT955459	KT955442	[58]
*Pt. javanica*	MFLUCC 11-0195	KJ742941	KT955460	KJ739606	[58]
*Pt. javanica*	MFLUCC 11-0159	KJ742940	KJ739607	KJ739605	[91]
*Quercicola fusiformis*	MFUCC 18-0479	MK348009	MK347898	MK360085	[83]
*Q. guttulospora*	MFUCC 18-0481	MK348010	MK347899	MK360086	[83]
*Xenoastrosphaeriella aquatica*	DLUCC 1525	MZ420753	MZ420755	MZ442701	[92]
*X. tornata*	MFLUCC 11-0196	KT955467	KT955447	KT955429	[58]
*X. trochus*	KUMCC 18-0194	MT659668	MT659669	MT653597	[93]

## 3. Results

### 3.1. Phylogenetic Analyses

**Figure 2:**
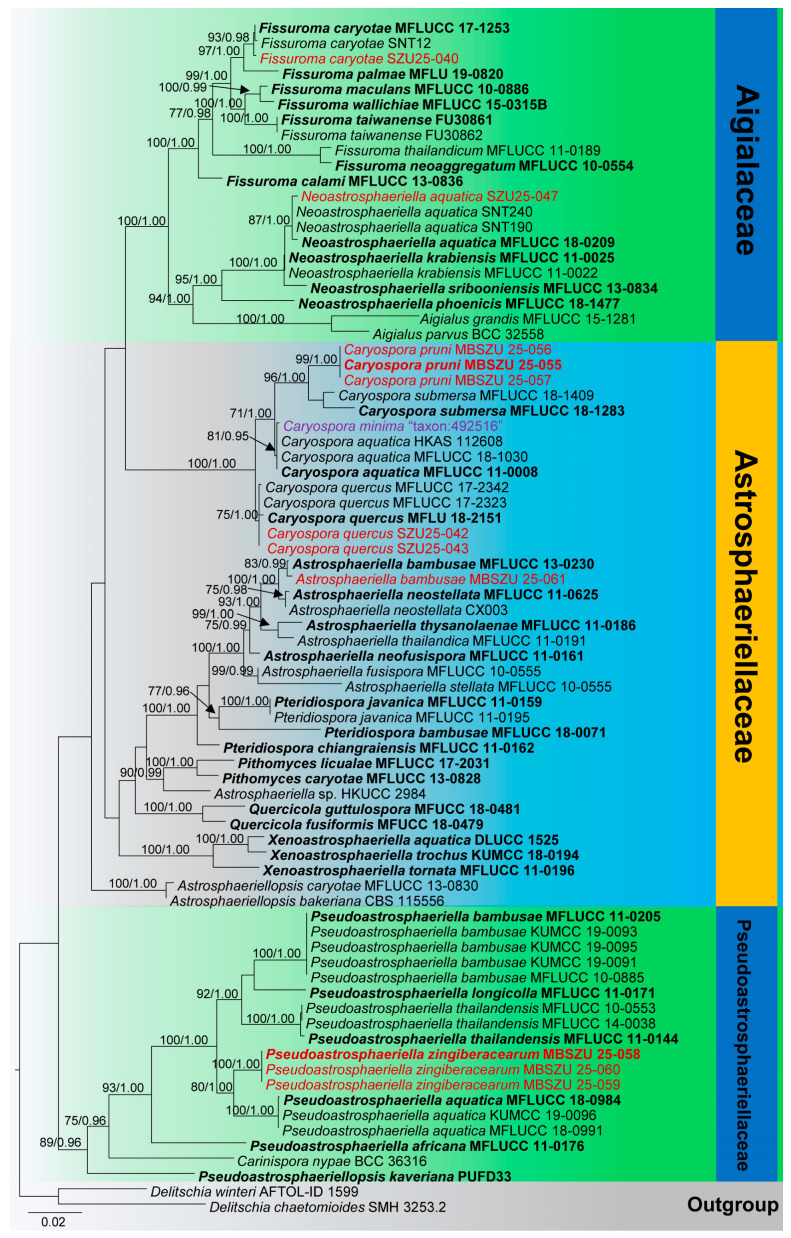
The phylogram generated from Maximum Likelihood analysis based on combined LSU, SSU, and *tef1-α* sequence data. The tree is rooted with *Delitschia chaetomioides* (SMH 3253.2) and *D. winteri* (AFTOL-ID 1599). The new isolates are in red, and ex-type strains are indicated in bold face. The proposed new combination is indicated in purple. Bootstrap support values ≥ 70% from the Maximum Likelihood (ML) and Bayesian posterior probabilities (BYPP) values ≥ 0.95 are given above the nodes, respectively.

The final combined gene (LSU, SSU, and *tef1-α*) data contained 78 strains (3268 characters with gaps), including newly added strains (SZU25-040 to SZU25-050). *Delitschia chaetomioides* (SMH 3253.2) and *D. winteri* (AFTOL-ID 1599) were used as outgroup taxa. Maximum Likelihood (ML) and Bayesian Inference (BI) analyses yielded congruent topologies, providing robust support to the inferred evolutionary relationships (Figure 2). The Bayesian analysis has resulted in 30,000 trees after 3,000,000 generations. Bootstrap support values for ML higher than 70% and BYPP greater than 0.90 are given above each branch, respectively (Figure 2). The overall topology of phylogenetic trees was concurred with previous studies [59,60,66]. Some other phylogenetic output data are mentioned in the Supplementary File S1.

Phylogenetic results indicate that isolate (SZU25-040) clusters with *Fissuroma* taxa, and makes a close phylogenetic relationship with *F. caryotae* isolates (MFLUCC 17-1253 and SNT 12); the isolate (SZU25-047) groups with *Neoastrosphaeriella aquatica* isolates (SNT 190, SNT 240, and MFLUCC 18-0209) in a well-supported clade (87% ML, 1.00 BYPP). Three isolates (MBSZU 25-055, MBSZU 25-056, and MBSZU 25-057) cluster together and make a sister lineage to *Caryospora submersa* isolates (MFLUCC 18-1283 and MFLUCC 18-1409); another two isolates (SZU25-042 and SZU25-043) also cluster with *Caryospora* species, but close to *C. quercus* isolates (MFLUCC 17-2323, MFLUCC 17-2342, and MFLU 18-2151). The isolate, MBSZU 25-061, groups with *Astrosphaeriella bambusae* (MFLUCC 13-0230) with robust statistical support (83% ML, 0.99 BYPP). In addition, another three isolates (MBSZU 25-058, MBSZU 25-059, and MBSZU 25-060) make a sister lineage with *Pseudoastrosphaeriella aquatica* isolates (MFLUCC 18-0984, MFLUCC 18-0991, and KUMCC 19-0096) with high statistical support (80% ML, 1.00 BYPP).

### 3.2. Taxonomy

#### 3.2.1. **Aigialaceae** Suetrong, Sakay., E.B.G. Jones, Kohlm., Volkm.-Kohlm. & C.L. Schoch, Stud. Mycol. 64: 166 (2009)

***Fissuroma*** Jian K. Liu, Phookamsak, E.B.G. Jones & K.D. Hyde, Fungal Diversity 51(1): 145 (2011)

This genus was introduced by Liu et al. [79] and typified by *Fissuroma maculans*, which was previously known as *Metasphaeria maculans* [94]. *Fissuroma* currently consists of 16 species, most of which have been recorded from terrestrial habitats [58,59,66,87]. Species have diverse morphological traits, such as ascomata with slit-like ostioles, trabeculate pseudoparaphyses, and fusiform, hyaline, 1-septate ascospores [79,88]. The asexual morph has been identified as coelomycetous, pleurophomopsis-like with globose to subglobose conidiomata, phialidic conidiogenous cells, and hyaline, globose, or oblong conidia [58,79,95,96]. The host specificity of *Fissuroma* species has yet to be investigated since they have been reported from various host species (e.g., *Arenga pinnata*, *Bambusa* spp., *Borassus flabellifer*, *Calamus andamanicus*, *Calamus conirostris*, *Calamus rotang*, *Caryota urens*, *Wallichia* sp.) [58,59,66,73,79,87,95].

***Fissuroma caryotae*** Wanas., E.B.G. Jones & K.D. Hyde, Mycological Progress 17(5): 579 (2018)

**Figure 3:**
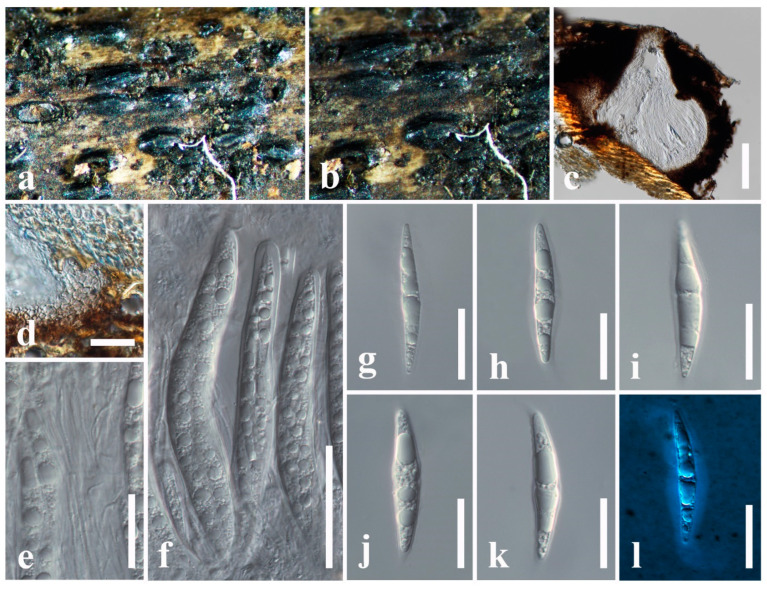
***Fissuroma caryotae*** (SZU25-040, **new host record**). (**a**,**b**) Appearance of ascomata on host surface. (**c**) Vertical section of ascoma. (**d**) Section through peridium. (**e**) Pseudoparaphyses. (**f**) Asci. (**g**–**k**) Ascospores. (**l**) Ascospore stained in Indian ink showing a mucilaginous sheath. Scale bars: (**c**) = 100 μm; (**d**,**g**–**l**) = 20 μm; (**e**,**f**) = 50 μm.

Index Fungorum number: IF554088; Facesoffungi number: FoF 03608; Figure 3

*Saprobic* on dead stem of *Arenga undulatifolia* (Arecaceae). **Sexual morph**: *Ascomata* 270–400 × 230–350 μm ($\bar{x}$ = 320 × 280 μm, *n* = 6), immersed or semi-immersed, appear as numerous, black or dark brown, raised, dome-shaped structures on host surface, solitary or clustered, hemispherical, flattened at the base, glabrous with rough walls, uni-loculate, carbonaceous, ostiolate. *Ostioles* central, with carbonaceous, slit-like opening. *Peridium* 15–42 μm wide ($\bar{x}$ = 28 μm, *n* = 10), carbonaceous, black, of unequal thickness, poorly developed at the base, thick at sides towards the apex, composed of several layers of brown to dark brown cells, inner layer composed of several layers of hyaline, *textura angularis* cells, thick-walled. *Hamathecium* comprising 1–2.5 μm wide ($\bar{x}$ = 1.9 μm, *n* = 10), septate, filiform, numerous, trabeculate pseudoparaphyses, embedded in a hyaline gelatinous matrix. *Asci* 100–170 × 12–15 μm ($\bar{x}$ = 130 × 13.8 μm, *n* = 20), 8-spored, bitunicate, fissitunicate, cylindrical to obclavate, apically rounded, short pedicellate, with an ocular chamber. *Ascospores* 37–48 × 6.4–9 μm ($\bar{x}$ = 41 × 7 μm, *n* = 30), overlapping, 1–2-seriate, hyaline, fusiform with acute ends, 1-septate, constricted at the septum, straight or slightly curved, asymmetrical, smooth-walled, with a thin mucilaginous sheath. **Asexual morph**: Undetermined.

Known hosts: *Arenga undulatifolia*, *Calamus* sp., and *Caryota urens* [59].

Known distribution: China and Thailand [59].

Material examined: China, Guangdong Province, Shenzhen, on dead stem of *Arenga undulatifolia* (Arecaceae), 15 January 2025, D.S. Tennakoon, DGSP040 (SZU25-040, new host record).

Notes: *Fissuroma caryotae* was introduced by Wanasinghe et al. [59] from a trunk of *Caryota urens* in China. The morphological characteristics of our collection (SZU25-040) strongly tally with the type of *F. caryotae* (MFLU 17-1253) in having immersed or semi-immersed, hemispherical, carbonaceous ascomata, trabeculate pseudoparaphyses, cylindrical to obclavate asci, and hyaline, fusiform, 1-septate ascospores with a thin mucilaginous sheath [59]. In addition, they share overlapping dimensions of asci (100–170 × 12–15 μm vs. 120–150 × 14–18 μm) and ascospores (37–48 × 6.4–9 μm vs. 40–50 × 7–9 μm). According to the phylogenetic analyses, our collection groups with *F. caryotae* isolates (MFLU 17-1253 and MFLUCC 16-1383) in a well-supported clade (93% ML, 0.98 BYPP). Thus, we identify our collection as a new host record of *F. caryotae* from *Arenga undulatifolia* in China.

#### 3.2.2. **Astrosphaeriellaceae** Phookamsak & K.D. Hyde, Fungal Diversity 74: 161 (2015)

***Astrosphaeriella*** Syd. & P. Syd., Annls Mycol. 11(3): 260 (1913)

*Astrosphaeriella* has a cosmopolitan distribution worldwide and is mostly reported from bamboos, palms, and grass species [58,60,66,95,97]. The typical morphological characteristics of *Astrosphaeriella* are conical to mammiform, carbonaceous ascomata, fissitunicate, cylindrical asci, and hyaline to brown, fusiform ascospores [58,93]. The asexual morph is coelomycetous and has phialidic, cylindrical to ampulliform conidiogenous cells and hyaline, globose to subglobose, aseptate conidia [58,95]. To date, 55 *Astrosphaeriella* species have been accepted [98].

***Astrosphaeriella bambusae*** Phookamsak & K.D. Hyde, Fungal Diversity: 165 (2015)

**Figure 4:**
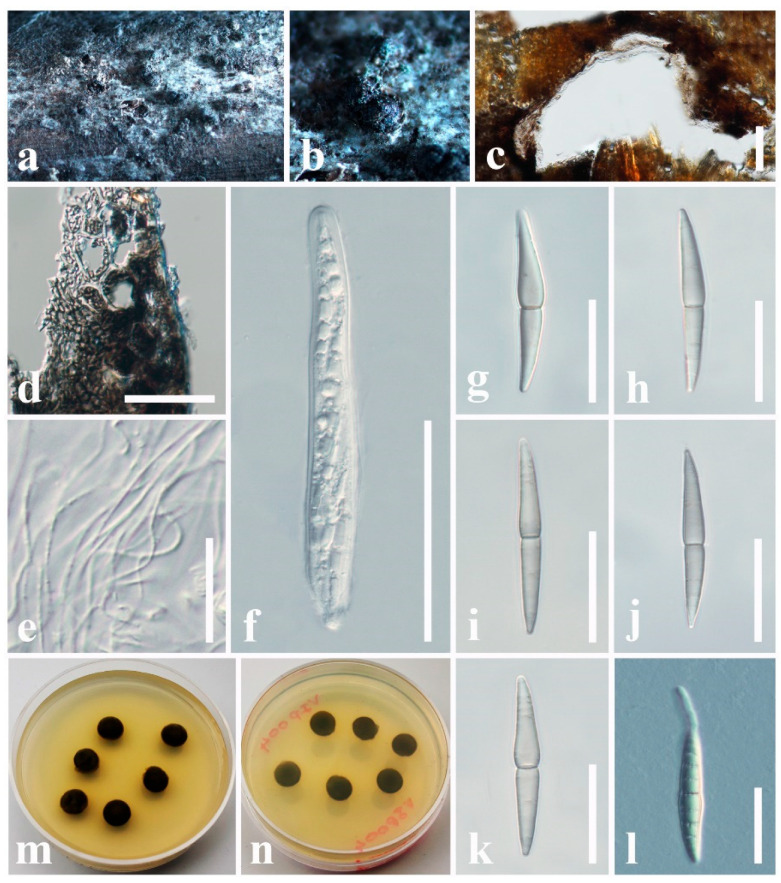
***Astrosphaeriella bambusae*** (SZU25-041, **new host record**). (**a**,**b**) Appearance of ascomata on host surface. (**c**) Vertical section of ascoma. (**d**) Section through peridium. (**e**) Pseudoparaphyses. (**f**) Ascus. (**g**–**k**) Ascospores. (**l**) Germinated ascospore. (**m**) Colony from above (in PDA). (**n**) Colony from below (in PDA). Scale bars: (**c**) = 100 μm; (**d**) = 15 μm; (**e**,**f**) = 80 μm; (**g**–**l**) = 20 μm.

Index Fungorum number: IF551633; Facesoffungi number: FoF 01223; Figure 4

*Saprobic* on dead stem of *Metroxylon sagu* (Arecaceae). **Sexual morph**: *Ascomata* 250–500 × 300–610 μm ($\bar{x}$ = 350 × 460 μm, *n* = 6), semi-immersed, erumpent through host surface, appear as numerous, black, raised, cone-like structures on host surface, solitary or clustered, mammiform to conical, flattened at the base, dark brown to black, uni-loculate, carbonaceous, with indistinct ostioles. *Peridium* 12–20 μm wide ($\bar{x}$ = 16 μm, *n* = 10), carbonaceous, black, of unequal thickness, poorly developed at the base, fragile, composed of several layers of brown to dark brown cells, thick-walled. *Hamathecium* comprising 1–2 μm wide ($\bar{x}$ = 1.6 μm, *n* = 10), septate, branched, filiform, numerous, trabeculate pseudoparaphyses, embedded in a hyaline gelatinous matrix. *Asci* 120–200 × 9–13 μm ($\bar{x}$ = 152 × 10 μm, *n* = 20), 8-spored, bitunicate, fissitunicate, cylindrical, short pedicellate, apically rounded, with an ocular chamber. *Ascospores* 32–40 × 5–6.4 μm ($\bar{x}$ = 35 × 5 μm, *n* = 30), overlapping, 1–2-seriate, hyaline when immature, pale brown at maturity, fusiform with acute ends, 1-septate, constricted at the septum, smooth-walled, without a mucilaginous sheath. **Asexual morph**: Undetermined.

Culture characteristics: Ascospores germinate on PDA within 8 h at 24 °C. Colonies on PDA reaching 10 mm diameter after 15 days at 24 °C. Colonies dense, circular, raised, surface with hyphae growing, rough-surface, fairly fluffy, with entire edge, margin well-defined. Colony from above: dark gray to dark brown at the center, and the margin; colony from below: dark brown to black at the center, and the margin.

Known hosts: Bamboo sp. and *Metroxylon sagu* [58,66]

Known distribution: China and Thailand [58,66]

Material examined: China, Guangdong Province, Shenzhen, on dead stem of *Metroxylon sagu* (Arecaceae), 12 January 2025, D.S. Tennakoon, DVIP004 (SZU25-041, new host record), living culture (MBSZU 25-061)

Notes: Due to morphological characteristics (e.g., semi-immersed to erumpent, mammiform to conical ascomata, trabeculate pseudoparaphyses, cylindrical, short pedicellate, apically rounded asci, and hyaline, 1-septate, fusiform ascospores) largely overlapping with *Astrosphaeriella bambusae* (MFLUCC 13-0230), we report our collection (MBSZU 25-061) as a new host record of *A. bambusae* from the dead stem of *Metroxylon sagu* in China. In particular, our collection shares overlapping dimensions of ascomata (250–500 × 300–610 μm vs. 360–600 × 380–660 μm), asci (120–200 × 9–13 μm vs. 130–180 × 10–12 μm), and ascospores (32–40 × 5–6.4 μm vs. 34–39 × 5–6 μm) with the type of *Astrosphaeriella bambusae* (MFLUCC 13-0230). Multi-gene phylogeny also indicates that our collection groups with *A. bambusae* (MFLUCC 13-0230) with a robust statistical support (83% ML, 0.99 BYPP). However, our collection differs from the *Astrosphaeriella bambusae* (MFLUCC 13-0230) by lacking a mucilaginous sheath. This is the first *Astrosphaeriella bambusae* species occurrence on *Metroxylon sagu* (Arecaceae).

***Caryospora*** De Not., Mém. R. Accad. Sci. Torino, Ser. 2 16: 463 (1857)

*Caryospora* was established by De Notaris [99] with *C. putaminum* as the type species. The taxonomic placement of *Caryospora* has been controversial for a long time. Initially, this was placed in Phaeophragmiae, based on the terminal septa and subsequently transferred to Zopfiaceae [100,101,102]. Ariyawansa et al. [80] transferred this genus to a new fungal family, Caryosporaceae, based on in-depth morphology and phylogeny evidence. However, this genus has been placed in Astrosphaeriellaceae in recent studies [8,60,83,103]. *Caryospora* species have been recorded from both terrestrial and freshwater habitats and characterized by obclavate asci with ellipsoidal to biconic ascospores [60,104]. To date, 12 *Caryospora* species have been accepted [98].

***Caryospora aquatica*** H. Zhang, K.D. Hyde & Ariyawansa, Fungal Diversity 75: 54 (2015), comb. nov.

Index Fungorum number: IF551418; Facesoffungi number: FoF 00958

Basionym: *Caryospora minima* Jeffers, Mycologia 32(4): 561 (1940)

Holotype: Berlin, Maryland, USA, putrescent putamina of Amygdalus persica, August 1938, BPI 71132.

Description: see Jeffers (1940)

*Caryospora minima* was introduced by Jeffers (1940) from MD, USA. Subsequently, Cai and Hyde [82] provided LSU and SSU sequence data (EU196550 and EU196551) for this species without any strain number/code, morphological description, herbarium details, or illustrations. In our multigene phylogenetic study, *Caryospora minima* strain (taxon:492516) was clustered with *C. aquatica* isolates (MFLUCC 11-0008, MFLUCC 18-1030, and HKAS 112608) in a well-supported clade (81% ML, 0.95 BYPP). The morphological characteristics of the type of *Caryospora minima* (BPI 71132) tally well with the type of *C. aquatica* (MFLUCC 11-0008) with overlapping characteristics and dimensions, such as hemispherical or conical ascomata (450–750 μm vs. 400–700 μm diam.), cylindrical clavate asci (160–190 × 60–80 μm vs. 150–180 × 35–50 μm), and 1-septete ascospores (44–52 × 20–27 μm vs. 40–50 × 20–30 μm) [80,100]. Thus, based on strong phylogenetic and morphological characteristics similarities, we treat *Caryospora minima* as a synonym of *C. aquatica* in this study.

***Caryospora pruni*** Tennakoon & S. Hongsanan, sp. nov.

**Figure 5:**
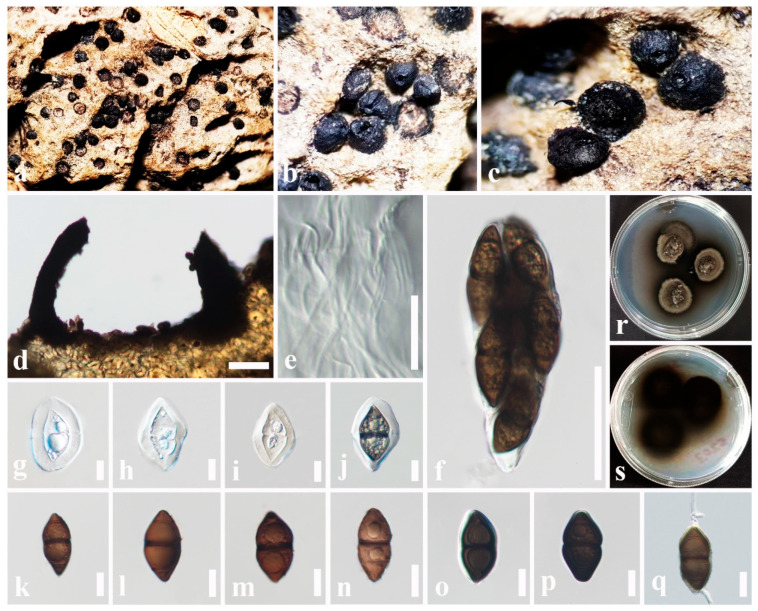
***Caryospora pruni*** (SZU25-044, **holotype**). (**a**) Appearance of ascomata on host surface. (**b**) Close-up of ascomata. (**c**,**d**) Vertical sections of ascomata. (**e**) Pseudoparaphyses. (**f**) Ascus. (**g**–**p**) Ascospores. (**q**) Germinated ascospore. (**r**) Colony from above (in PDA). (**s**) Colony from below (in PDA). Scale bars: (**d**) = 100 μm; (**e**,**f**) = 50 μm; (**g**–**q**) = 20 μm.

Index Fungorum number: IF904313; Facesoffungi number: FoF 18109; Figure 5

Etymology: The specific epithet “pruni” was given after the host genus.

Holotype: SZU25-044

*Saprobic* on decaying fruit pericarp of *Prunus persica* (Rosaceae). **Sexual morph**: *Ascomata* 350–650 × 400–500 μm ($\bar{x}$ = 490 × 350 μm, *n* = 6), superficial, appear as numerous, black, raised, cone-like structures on host surface, solitary or clustered, conical, flattened at the base, dark brown to black, uni-loculate, carbonaceous, ostiolate. *Ostiole* central, apapillate, carbonaceous, not prominent. *Peridium* 18–40 μm wide ($\bar{x}$ = 26 μm, *n* = 10), carbonaceous, black, poorly developed at the base, fragile, composed of several layers of brown to dark brown cells, thick-walled. *Hamathecium* comprising 1–2 μm wide ($\bar{x}$ = 1.5 μm, *n* = 10), septate, branched, filiform, numerous, trabeculate pseudoparaphyses. *Asci* 95–120 × 33–50 μm ($\bar{x}$ = 108 × 41 μm, *n* = 20), 8-spored, bitunicate, fissitunicate, cylindric-clavate, short pedicellate, apically rounded with an ocular chamber. *Ascospores* 45–56 × 20–30 μm ($\bar{x}$ = 51 × 26 μm, *n* = 30), overlapping, 1–2-seriate, hyaline when immature, pale brown to dark brown at maturity, ellipsoidal, diamond-shaped, apex pointed, 1-septate, distinctly constricted at the septum, mostly with a dark band around the septum, walls thickened at both ends, symmetrical or asymmetrical, straight or slightly curved, guttulate, present polar germ pores, surrounded by a gelatinous mucilaginous sheath when immature (5–8 μm wide). **Asexual morph**: Undetermined.

Culture characteristics: Ascospores germinated on PDA within 8 h at 24 °C. Colonies on PDA reached 21 mm in diameter after 12 days at 24 °C. Colonies dense, circular, raised, surface with hyphae growing, smooth-surface, fairly fluffy, with entire edge, margin well-defined. Colony from above: dark gray to dark brown at the center, gray at the margin; colony from below: dark brown to black at the center, pale brown at the margin.

Material examined: China, Guangdong Province, Shenzhen, on decaying fruit pericarp of *Prunus persica* (Rosaceae), 17 January 2025, D.S. Tennakoon, SZ3 (SZU25-044, holotype), ex-type living culture (MBSZU 25-055); ibid. 18 January 2025, DPSZ3B, DPSZ3 (SZU25-045, SZU25-046, isotypes), living cultures (MBSZU 25-056, MBSZU 25-057).

Notes: The morphological characteristics of our collection resemble generic concept of *Caryospora* in having conical, uni-loculate, carbonaceous ascomata, cylindric-clavate, short pedicellate asci, and ellipsoidal, diamond-shaped ascospores [60,80,83,104]. Multi-gene phylogeny revealed that our isolates (MBSZU 25-055, MBSZU 25-056, and MBSZU 25-057) form an independent lineage sister to *Caryospora submersa* isolates (MFLUCC 18-1283 and MFLUCC 18-1409) with 96% ML and 1.00 BYPP support. However, our collection can be distinguished from *C. submersa*, which has broadly cylindrical to clavate asci and pale brown to dark brown, diamond-shaped ascospores with polar germ pores, whereas *C. submersa* has obclavate asci and narrowly fusiform, hyaline, thin-walled ascospores [60]. Thus, we introduce our collection as a new species, *Caryospora pruni* from *Prunus persica* in China.

***Caryospora quercus*** Jayasiri, E.B.G. Jones & K.D. Hyde, Mycosphere 10(1): 34 (2019)

**Figure 6:**
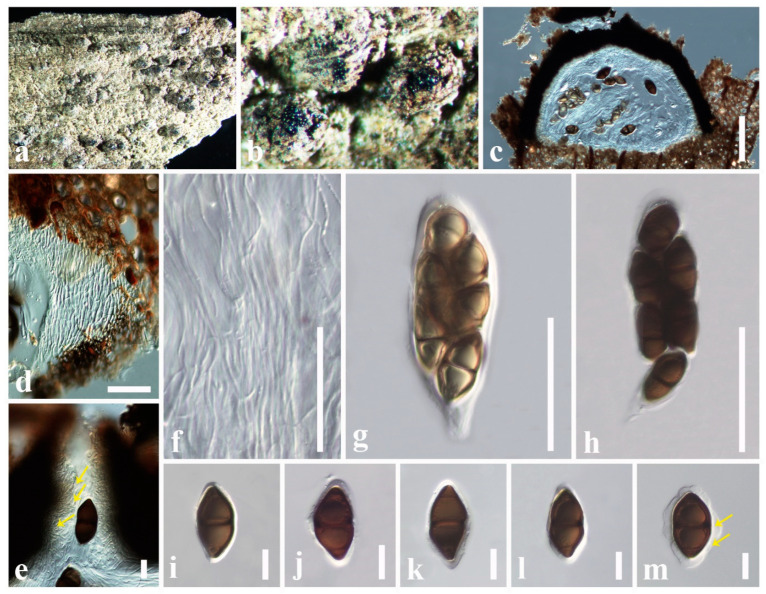
***Caryospora quercus*** (SZU25-042, **new host record**). (**a**) Appearance of ascomata on host surface. (**b**) Close-up of ascomata. (**c**) Vertical section of ascoma. (**d**) Section through peridium. (**e**) Section through ostiole (arrows show periphyses). (**f**) Pseudoparaphyses. (**g**,**h**) Asci. (**i**–**m**) Ascospores (arrows show mucilaginous sheath). Scale bars: (**c**) = 100 μm; 1 (**d**) = 5 μm; (**e**,**i**–**m**) = 20 μm; (**f**–**h**) = 80 μm.

Index Fungorum number: IF555535; Facesoffungi number: FoF 05236; Figure 6

*Saprobic* on dead stem of *Citrus maxima* (Rutaceae). **Sexual morph**: *Ascomata* 250–400 × 300–520 μm ($\bar{x}$ = 290 × 450 μm, *n* = 6), semi-immersed, erumpent, appear as black dots, solitary or clustered, conical, flattened at the base, dark brown to black, uni-loculate, carbonaceous, ostiolate. *Ostiole* central and filled with periphyses. *Peridium* 10–15 μm wide ($\bar{x}$ = 13 μm, *n* = 10), carbonaceous, black, poorly developed at the base, fragile, two-layered, outer layer strongly carbonized, composed of several layers of brown to dark brown cells, inner layer composed of several layers of hyaline cells, thick-walled. *Hamathecium* comprising 1–2 μm wide ($\bar{x}$ = 1.5 μm, *n* = 10), septate, branched, filiform, numerous, trabeculate pseudoparaphyses. *Asci* 90–200 × 25–38 μm ($\bar{x}$ = 145 × 32 μm, *n* = 20), 8-spored, bitunicate, fissitunicate, cylindric-clavate, short pedicellate, apically rounded with an ocular chamber. *Ascospores* 40–50 × 16–30 μm ($\bar{x}$ = 46 × 22 μm, *n* = 30), overlapping, 1–2-seriate, hyaline when immature, pale brown to dark brown at maturity, ellipsoidal, diamond-shaped, apex pointed, 1-septate, distinctly constricted at the septum, mostly with a dark band around the septum, walls thickened at both ends, symmetrical or asymmetrical, guttulate, present polar germ pores, surrounded by a gelatinous mucilaginous sheath (5–8 μm wide). **Asexual morph**: Undetermined.

Known hosts: *Citrus maxima* and *Quercus* sp. (Jayasiri et al., 2019 [83]; Dong et al., 2020 [60]; this study)

Known distribution: China and Thailand (Jayasiri et al., 2019 [83]; Dong et al., 2020 [60]; this study)

Material examined: China, Guangdong Province, Shenzhen, on dead stem of *Citus maxima* (Rutaceae), 12 January 2025, D.S. Tennakoon, WD002 (SZU25-042, new host record), ibid. 15 January 2025, DST5 (SZU25-043).

Notes: According to the multi-gene phylogeny (LSU, SSU, and *tef1-α*), our collection (SZU25-042 and SZU25-043) clustered with *Caryospora quercus* isolates (MFLUCC 17-2323, MFLUCC 17-2342, and MFLU 18–2151) in a well-supported clade (75% ML, 1.00 BYPP). Morphological characteristics also indicate that our collection resembles *C. quercus* in having conical, dark brown to black, uni-loculate, carbonaceous ascomata, cylindric-clavate, short pedicellate asci, and ellipsoidal, diamond-shaped, 1-septate ascospores with gelatinous mucilaginous sheath [60,83,93]. In addition, our collection has overlapping dimensions of ascomata (250–400 × 300–520 μm vs. 300–420 × 450–483 μm), asci (90–200 × 25–38 μm vs. 110 –147 × 30–35 μm), and ascospores (40–50 × 16–30 μm vs. 41–54 × 18–28 μm) as well [83]. Based on the robust morphology and phylogeny support, we identify our collection as *C. quercus*. *Caryospora quercus* was initially introduced by Jayasiri et al. [83] from decaying fruit pericarp of *Quercus* sp. (Fagaceae) in Thailand. Thus, we introduce our collection as a new host record of *C. quercus* from *Citrus maxima* in China.

***Neoastrosphaeriella*** Jian K. Liu, E.B.G. Jones & K.D. Hyde, Fungal Diversity 51(1): 148 (2011)

Liu et al. [79] introduced *Neoastrosphaeriella* to accommodate *N. krabiensis* as the type species, which was collected from a dead petiole of *Metroxylon sagu*. The species of *Neoastrosphaeriella* have been recorded from both terrestrial and freshwater habitats [59,79,86,89,105]. In particular, most have been reported from Thailand (*N. aquatica*, *N. krabiensis*, *N. phoenicis*, and *N. sribooniensis*). *Neoastrosphaeriella* species are characterized by immersed to semi-immersed ascomata with slit-like ostioles, fissitunicate, obclavate asci, and brown, verrucose ascospores [79]. To date, there are five *Neoastrosphaeriella* species, such as *N. alankrithabeejae*, *N. aquatica*, *N. krabiensis*, *N. phoenicis*, and *N. sribooniensis* [98].

***Neoastrosphaeriella aquatica*** D.F. Bao, Z.L. Luo, K.D. Hyde & Hong Y. Su, Phytotaxa 391(3): 201 (2019)

**Figure 7:**
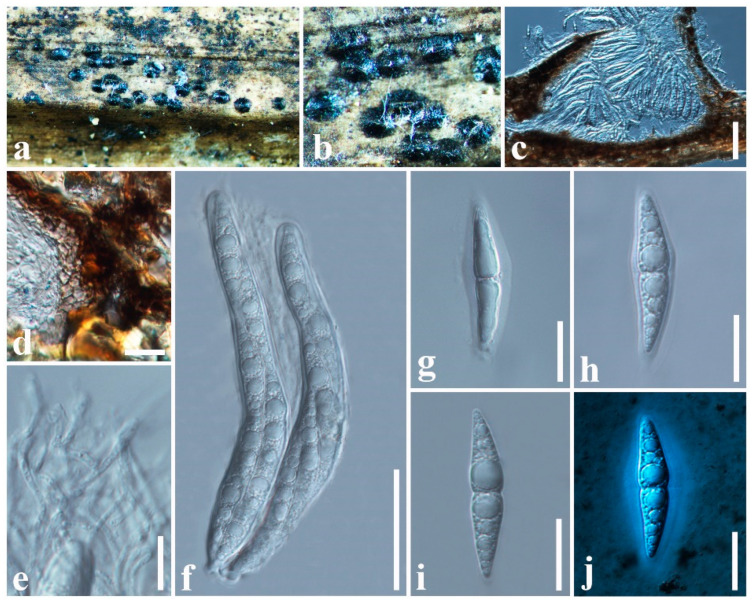
***Neoastrosphaeriella aquatica*** (SZU25-047, **new host record**). (**a**,**b**) Appearance of ascomata on host surface. (**c**) Vertical section of ascoma. (**d**) Section through peridium. (**e**) Pseudoparaphyses. (**f**) Asci. (**g**–**i**) Ascospores. (**j**) Ascospore stained in Indian ink showing a mucilaginous sheath. Scale bars: (**c**) = 100 μm; (**d**) = 20 μm; (**e**,**f**) = 30 μm; (**g**–**j**) = 15 μm.

Index Fungorum number: IF555357; Facesoffungi number: FoF 04910; Figure 7

*Saprobic* on dead stem of *Phoenix paludosa* (Arecaceae). **Sexual morph**: *Ascomata* 210–520 × 200–350 μm ($\bar{x}$ = 341 × 230 μm, *n* = 6), semi-immersed, appear as numerous, black or dark brown, raised, dome-shaped structures on host surface, solitary or clustered, hemispherical with wedged sides, flattened at the base, glabrous with rough walls, uni-loculate, carbonaceous, ostiolate. *Ostioles* central, with carbonaceous, slit-like opening. *Peridium* 16–28 μm wide ($\bar{x}$ = 23 μm, *n* = 10), carbonaceous, black, of unequal thickness, poorly developed at the base, thick at sides towards the apex, composed of several layers of brown to dark brown cells, inner layer composed of several layers of hyaline, *textura angularis* cells, thick-walled. *Hamathecium* comprising 1–2 μm wide ($\bar{x}$ = 1.6 μm, *n* = 10), septate, filiform, numerous, cellular pseudoparaphyses, embedded in a hyaline gelatinous matrix. *Asci* 80–100 × 12–16 μm ($\bar{x}$ = 92 × 14 μm, *n* = 20), 8-spored, bitunicate, fissitunicate, cylindrical to obclavate, apically rounded, with a short furcate pedicel, with an ocular chamber. *Ascospores* 30–40 × 5–7 μm ($\bar{x}$ = 33 × 6 μm, *n* = 30), overlapping, 1–2-seriate, hyaline, fusiform with acute ends, 1-septate, constricted at the septum, straight or slightly curved, asymmetrical, smooth-walled, guttulate, with a distinct mucilaginous sheath. **Asexual morph**: Undetermined.

Known hosts: decaying submerged wood of unknown host, and *Phoenix paludosa* [89].

Known distribution: China and Thailand [89]

Material examined: China, Guangdong Province, Shenzhen, on dead stem of *Phoenix paludosa* (Arecaceae), 20 January 2025, D.S. Tennakoon, DPC044 (SZU25-047, new host record).

Notes: In this study, our phylogenetic analyses indicate that our collection (SZU25-047) groups with *Neoastrosphaeriella aquatica* isolates (MFLUCC 18–0209, MFLUCC 18-1531, HKAS 105481) in a well-supported clade (87% ML, 1.00 BYPP). The morphological characteristics also tally well with the type of *N. aquatica* in having semi-immersed, solitary or clustered, hemispherical ascomata with slit-like ostioles, cylindrical to obclavate asci, and hyaline, fusiform, 1-septate ascospores [89]. Their overlapping dimensions of ascomata (210–520 × 200–350 μm vs. 250–400 × 160–250 μm), asci (80–100 × 12–16 μm vs. 84–112 × 14–19 μm), and ascospores (30–40 × 5–7 μm vs. 31–37 × 5–8 μm) also indicate that they are the same species. Therefore, based on both morphology and phylogeny support, we report our collection as a new host record of *N. aquatica* from China.

#### 3.2.3. **Pseudoastrosphaeriellaceae** Phookamsak & K.D. Hyde, Fungal Diversity: 181 (2015)

***Pseudoastrosphaeriella*** Phookamsak, Z.L. Luo & K.D. Hyde, Fungal Diversity: 182 (2015)

Phookamsak et al. [58] introduced *Pseudoastrosphaeriella* to accommodate astrosphaeriella-like species in Pseudoastrosphaeriellaceae. *Pseudoastrosphaeriella* was typified by *P. thailandensis*, which was collected from a dead stem of bamboo in Thailand. The morphology of *Pseudoastrosphaeriella* species differs from *Astrosphaeriella* by ascomata characteristics, such as immersed, hemispherical or dome-shaped with short to long necks, whereas *Astrosphaeriella* has erumpent, raised, conical ascomata and has a star-like or rounded flange with small papilla [58]. Currently, seven *Pseudoastrosphaeriella* species have been accepted, such as *P. aequatoriensis*, *P. africana*, *P. aquatica*, *P. bambusae*, *P. longicolla*, *P. papillate*, and *P. thailandensis* [98].

***Pseudoastrosphaeriella zingiberacearum*** Tennakoon & S. Hongsanan, sp. nov.

**Figure 8:**
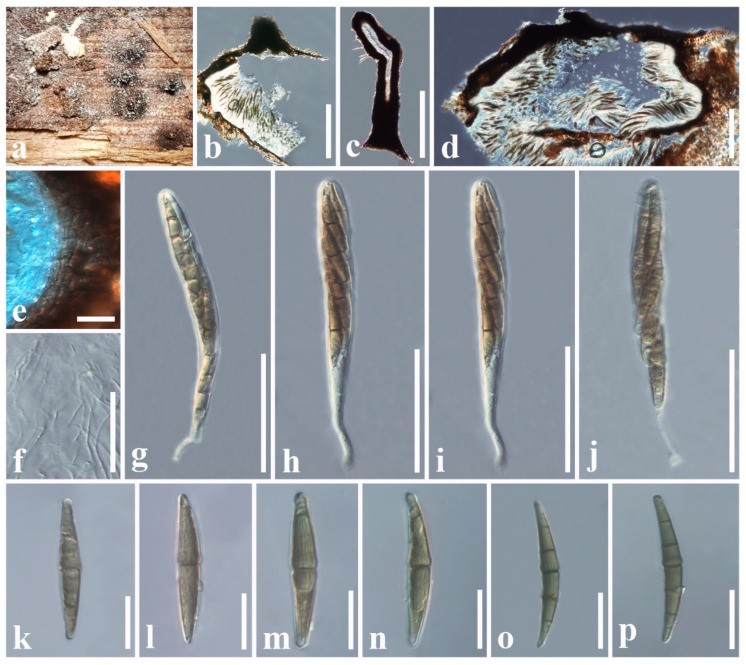
***Pseudoastrosphaeriella zingiberacearum***(SZU25-048, **holotype**). (**a**) Appearance of ascomata on host surface. (**b**,**d**) Vertical section of ascoma. (**c**) Section through neck. (**e**) Section through peridium. (**f**) Pseudoparaphyses. (**g**–**j**) Asci. (**k**–**p**) Ascospores. Scale bars: (**b**,**d**) = 200 μm; (**c**,**k**–**p**) = 60 μm; (**e**) = 20 μm; (**f**–**j**) = 70 μm.

Index Fungorum number: IF904314; Facesoffungi number: FoF 18110; Figure 8

Etymology. The specific epithet zingiberacearum was given after the host family, Zingiberaceae.

Holotype: SZU25-048

*Saprobic* on dead stem of *Hedychium coronarium* (Zingiberaceae). **Sexual morph**: *Ascomata* 400–600 × 800–1300 μm ($\bar{x}$ = 520 × 980 μm, *n* = 6) (excluding neck), immersed in host epidermis, erumpent through host surface, appear as numerous, black or dark brown, raised, dome-shaped structures on host surface, solitary or clustered, uni-loculate, carbonaceous, depressed conical or lageniform, flattened at the base, ostiolate. *Necks* 100–200 × 5–10 μm ($\bar{x}$ = 125 × 8 μm, *n* = 4), dark brown or black, central, cylindrical, oblique, fragile, carbonaceous. *Peridium* 14–38 μm wide ($\bar{x}$ = 26 μm, *n* = 10), carbonaceous, black, of unequal thickness, poorly developed at the base, thick at sides towards the apex, composed of several layers of brown to dark brown cells, comprising host cells plus fungal tissue at outside, inner layer composed of several layers of pale brown or hyaline, *textura angularis* cells, thick-walled. *Hamathecium* comprising 1–2.6 μm wide ($\bar{x}$ = 1.8 μm, *n* = 10), septate, filiform, numerous, trabeculate pseudoparaphyses, anastomosing at the apex, embedded in a hyaline gelatinous matrix. *Asci* 150–172 × 11–14 μm ($\bar{x}$ = 160 × 12 μm, *n* = 20), 8-spored, bitunicate, fissitunicate, cylindrical to clavate, apically rounded, with a short furcate pedicel, with an ocular chamber. *Ascospores* 46–57 × 5–7 μm ($\bar{x}$ = 52 × 6 μm, *n* = 30), overlapping, 1–2-seriate, initially hyaline, pale brown to brown when mature, broadly fusiform with acute or rounded ends, 1–6-septate, constricted at the middle septum, straight or slightly curved, asymmetrical, slightly swollen above middle septum, rough-walled with minute striations, guttulate. **Asexual morph**: Undetermined.

Material examined: China, Jiangxi Province, Nanchang, on dead stem of *Hedychium coronarium* (Zingiberaceae), 20 January 2024, D.S. Tennakoon, DDP007 (SZU25-048, holotype), ex-type living culture (MBSZU 25-058); ibid. 29 January 2025, DP007-ITB, DP007-ITC (SZU25-049, SZU25-050, isotypes), living cultures (MBSZU 25-059, MBSZU 25-060).

Notes: In our phylogenetic analysis, our collection (MBSZU 25-058, MBSZU 25-059, and MBSZU 25-060) nested with *Pseudoastrosphaeriella* isolates and formed a sister lineage with *P. aquatica* with 80% ML and 1.00 BYPP statistical support (Figure 2). The morphological characteristics of our collection resemble *Pseudoastrosphaeriella* in having hemispherical ascomata, cylindrical to clavate, apically rounded asci, and reddish brown ascospores, with minute striations [58,60]. Our collection can be distinguished from *P. aquatica* in having larger ascomata (400–600 × 800–1300 μm vs. 250–350 × 280–320 μm), short neck (100–200 × 5–10 μm vs. 450–500 × 100–110 μm), narrow asci (150–172 × 11–14 μm vs. 145–260 × 19–25 μm), and broadly fusiform, 1–6-septate ascospores with striations (46–57 × 5–7 μm vs. 40–48 × 8–10.5 μm), whereas *P. aquatica* has 3-septate ascospores with prominent guttules and lacking striations [60]. In addition, we compared our collection (MBSZU 25-058) with *P. aquatica* (MFLUCC 18-0984) based on base pair differences, and there are 39 base pair differences (4.67%) across 834 nucleotides in the *tef1-α* gene region. Thus, due to these phylogenetic and morphological distinctions, we have introduced our collection as a novel species, *Pseudoastrosphaeriella zingiberacearum*, collected from *Hedychium coronarium* in China.

### 3.3. Geographical Distribution and Their Host Occurrences of Aigialaceae, Astrosphaeriellaceae, and Pseudoastrosphaeriellaceae Species

**Figure 9:**
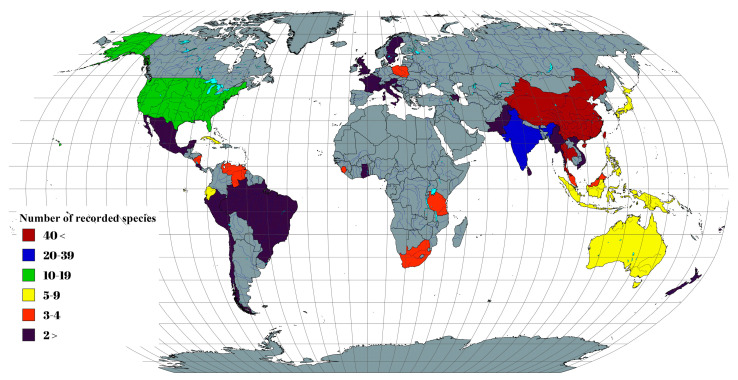
Distribution of so-far reported species in Aigialaceae, Astrosphaeriellaceae, and Pseudoastrosphaeriellaceae worldwide. Color gradient shows the number of recorded species from lowest (purple) to highest (maroon) and no records (gray).

This study documents 164 species across Aigialaceae, Astrosphaeriellaceae, and Pseudoastrosphaeriellaceae, representing 18 genera, along with their host associations and geographical distributions (Table 3). Based on the data collected, it appears that the members of Aigialaceae, Astrosphaeriellaceae, and Pseudoastrosphaeriellaceae have mostly been collected from China (46 records) and Thailand (41 records). This could be attributed to the well-documented fungal collections, coupled with the presence of specialized research institutions and proper identification techniques (e.g., morphology coupled with multi-gene phylogenetic analyses, fungal evolution analyses, Genealogical Concordance Phylogenetic Species Recognition (GCPSR) analysis to distinguish the cryptic species) in these countries. Apart from these main countries, significant records exist from India (22 records), the United States (12 records), Brunei (11 records), Australia (9 records), Indonesia and Japan (8 records), Ecuador (7 records), Papua New Guinea (6 records), Cuba and Philippines (5 records), while other have less than five species records from 28 countries (e.g., Brazil, France, Italy, Malaysia, Nicaragua, Pakistan, Peru, Poland, South Africa, Sri Lanka, Sweden, Tanzania, Venezuela, Vietnam, etc.) (Figure 9).

**Figure 10:**
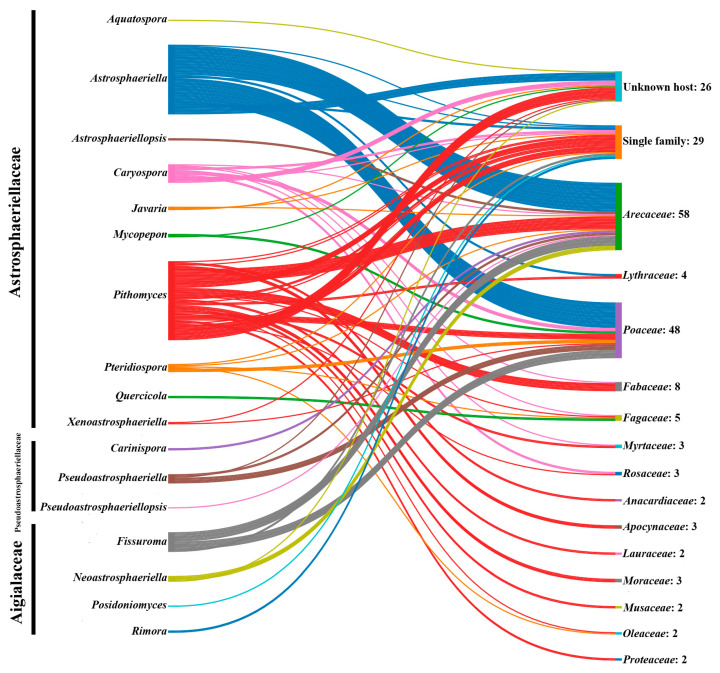
The Aigialaceae, Astrosphaeriellaceae, and Pseudoastrosphaeriellaceae species distribution through plant host families.

**Figure 11:**
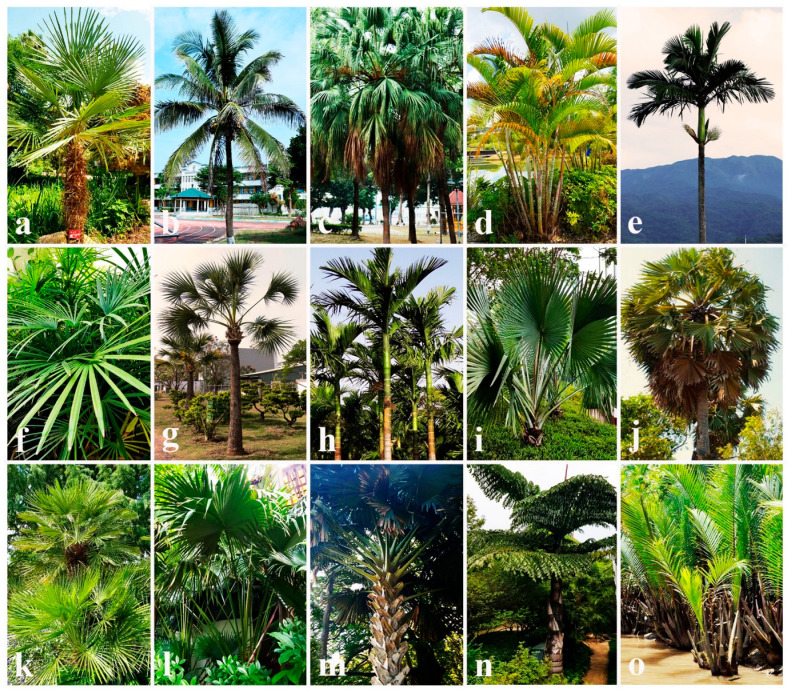
Common Arecaceae species that have reported Aigialaceae, Astrosphaeriellaceae, and Pseudoastrosphaeriellaceae species. (**a**) *Trachycarpus princeps*. (**b**) *Cocos nucifera*. (**c**) *Livistona chinensis*. (**d**) *Chrysalidocarpus lutescens*. (**e**) *Archontophoenix alexandrae*. (**f**) *Rhapis Excelsa*. (**g**) *Sabal palmetto*. (**h**) *Areca catechu*. (**i**) *Bismarckia nobilis*. (**j**) *Borassus flabellifer*. (**k**) *Chamaerops humilis*. (**l**,**m**) *Corypha umbraculifera*. (**n**) *Caryota obtusa*. (**o**) *Nypa fruticans*.

A key finding reveals that Arecaceae (58 records) and Poaceae (48 records) represent the predominant host families for these fungal taxa (Figure 10). This raises an exciting ecological question: Are these species host-specific to either the Arecaceae or Poaceae families? (Figure 10 and Figure 11). In addition, these species produce carbonaceous fruiting bodies, raising questions about potential relationships between their nutritional or evolutionary adaptations and their Arecaceae/Poaceae hosts. In particular, these interactions are critical for understanding biodiversity, ecosystem functioning, and the evolutionary adaptations of these fungi to their environments. However, this could be answered with a comprehensive study with multiple taxon samplings. The geographical distribution and host occurrences of each family are discussed in the following section.

#### 3.3.1. Aigialaceae

In total, 29 Aigialaceae species in five genera (*Aigialus*, *Fissuroma*, *Neoastrosphaeriella*, *Posidoniomyces*, and *Rimora*) are listed with their geographical distributions and host occurrences (Table 3). According to the collected data, Aigialaceae species have been reported from subtropical, tropical, and temperate countries worldwide (e.g., Australia, Brunei, China, Croatia, Ecuador, India, Japan, Thailand, Papua New Guinea, the Philippines, and the United States). Of them, the highest number of species records has been reported from Thailand (12 records), and this is followed by India (7 records), China (5 records), and the United States (3 records), while other countries have single records (Table 3). It is worth noting that the majority of Aigialaceae species have been recorded from Asia. Some species exhibit extensive distribution throughout multiple countries; for instance, *Fissuroma fissuristomum* has been reported in Australia, Brunei, China, Ecuador, and Papua New Guinea [73,106,107]. The lower number of species recorded may indicate that fewer taxonomic investigations have been conducted in those countries.

When considering the host occurrences of Aigialaceae species, most of the species have been recorded from Arecaceae host species (12 records), such as *Arenga pinnata*, *Borassus flabellifer*, *Calamus andamanicus*, *C. conirostris*, *C. rotang*, *Caryota urens*, *Metroxylon sagu*, and *Phoenix paludosa* (Table 3). In addition, some others have been reported from Poaceae hosts (7 records; *Bambusa* spp. and *Phyllostachys reticulata*) and Rhizophoraceae host species (6 records; *Rhizophora apiculata*, *R. mangle*, and *R. mucronata*). In particular, the occurrence of Rhizophoraceae host species is interesting since they have been collected from submerged root parts (e.g., *Aigialus grandis*, *A. mangrovis*, *A. parvus*, *A. rhizophorae*, and *A. striatisporus*). Other species’ host occurrence has been limited to single host families, such as Lythraceae, Posidoniaceae, Theaceae, Verbenaceae, and Zingiberaceae (Table 3).

#### 3.3.2. Astrosphaeriellaceae

In total, 126 Astrosphaeriellaceae species in 10 genera (*Aquatospora*, *Astrosphaeriella*, *Astrosphaeriellopsis*, *Caryospora*, *Javaria*, *Mycopepon*, *Pithomyces*, *Pteridiospora*, *Quercicola*, and *Xenoastrosphaeriella*) are listed with their geographical distributions and host occurrences (Table 3). Our summarized data suggests that Astrosphaeriellaceae species exhibit a cosmopolitan distribution, given their presence on numerous continents (e.g., Asia, Africa, Australia, Europe, North America, and South America). Of them, the majority of species records have been reported from Asian countries, such as Brunei, China, India, Indonesia, Japan, the Philippines, and Thailand (Table 3). The highest number of species have been collected from China (42 records), and this is followed by Thailand (24 records), India (14 records), the United States (9 records), Indonesia (8 records), Australia and Japan (7 records), Brunei (6 records), Cuba, Ecuador and Papua New Guinea (5 records), Nicaragua, Philippines, Poland, Sierra Leone, Tanzania, and South Africa (3 records) and other have less than two species records (e.g., Austria, Brazil, Costa Rica, France, Ghana, Italy, Malaysia, Mexico, Myanmar, New Zealand, Pakistan, Peru, Seychelles, Singapore, Sri Lanka, Sweden, the United Kingdom, and Vietnam). Some species, like *Astrosphaeriella stellata*, are widely distributed (e.g., Australia, China, India, Japan, Papua New Guinea, the Philippines, and Vietnam). In addition, *Astrosphaeriella vesuvius* has been reported from eight countries, including Australia, Brunei, Indonesia, Thailand, Malaysia, Papua New Guinea, and Sri Lanka. The substantial number of species records from China, India, and Thailand may result from extensive taxonomic sampling and in-depth studies conducted in these countries over the last two decades [58,59,60,83,90,105,108,109,110].

The host associations of Astrosphaeriellaceae species are particularly noteworthy, with documented occurrences across 37 different plant families (Table 3). Of them, highest numbers of species records have been reported from Arecaceae (41 records) and followed by Poaceae (36 records), Fagaceae (13 records), Lythraceae (4 records), Apocynaceae, Moraceae, Myrtaceae, and Rosaceae (3 records), Anacardiaceae, Lauraceae, Musaceae, Oleaceae, Proteaceae (2 records), and others have single occurrences (e.g., Acoraceae, Altingiaceae, Annonaceae, Bromeliaceae, Cannaceae, Juglandaceae, Juncaceae, Liliaceae, Malvaceae, Sapindaceae) (Table 3). The most frequently recorded Arecaceae hosts include *Archontophoenix alexandrae*, *Arenga undulatifolia*, *Calamus* spp., *Cocos nucifera*, *Daemonorops margaritae*, *Eleiodoxa conferta*, *Licuala longicalycata*, *Livistona* spp., *Nothofagus* spp., *Oncosperma* spp., and *Sabal palmetto* (Table 3). The most commonly reported Poaceae hosts are Bambusa spp., *Miscanthus* spp., *Melocalamus compactiflorus*, *Phyllostachys* spp., and *Saccharum officinarum* [73]. Several species, including *Pithomyces cupaniae*, exhibit broad host ranges in multiple plant families such as Anacardiaceae, Anisophylleaceae, Apocynaceae, Fabaceae, Lauraceae, Oleaceae, and Sapindaceae [73,111,112]. Furthermore, both *Pithomyces graminicola* (associated with Anacardiaceae, Cannaceae, Fabaceae, Lauraceae, Poaceae, and Strelitziaceae) and *P. obscuriseptatus* (associated with Acoraceae, Arecaceae, Butomaceae, Cyperaceae, Juncaceae, and Typhaceae) exhibit extensive host ranges [73,110,113,114,115,116]. Another interesting fact is that some Astrosphaeriellaceae species have been collected from soil as well. Beyond their plant associations, some Astrosphaeriellaceae species (e.g., P. *longiclavisporus* and *P. pallidus*) have been collected from soil habitats in China [117].

#### 3.3.3. Pseudoastrosphaeriellaceae

In total, 10 Pseudoastrosphaeriellaceae species in three genera (*Carinispora*, *Pseudoastrosphaeriella*, and *Pseudoastrosphaeriellopsis*) are listed with their geographical distributions and host occurrences (Table 3). Current records indicate Pseudoastrosphaeriellaceae species have been mostly collected from Thailand (5 records) and Brunei (4 records), with single occurrences reported from Australia, Ecuador, India, Malaysia, Philippines, and Tanzania [58,73,118,119]. Reported species have been mainly associated with Arecaceae hosts (*Arenga undulatifolia*, *Avicennia marina*, *Calamus* spp., *Daemonorops* spp., *Licuala longicalycata*, *Nypa fruticans*, *Oncosperma tigillarium*, and *Phytelephas* spp.) and Poaceae hosts (*Bambusa* spp. and *Phragmites australis*) (Table 3).

**Table 3:** Host association and geographical distribution of reported Aigialaceae, Astrosphaeriellaceae, and Pseudoastrosphaeriellaceae species.

**Species**	**Host**	**Family**	**Locality**	**References**
*Aigialus grandis*	*Rhizophora mangle*, *Rhizophora mucronata*, *Sonneratia acida*	Lythraceae, Rhizophoraceae	The United States, India	[120,121,122]
*Aigialus mangrovis*	*Rhizophora mucronata*	Rhizophoraceae	India	[121,123]
*Aigialus parvus*	*Avicennia alba*, *Rhizophora mangle*, *Sonneratia acida*	Lythraceae, Rhizophoraceae	The United States	[121,122]
*Aigialus rhizophorae*	*Rhizophora mucronata*	Rhizophoraceae	India	[121,123]
*Aigialus striatisporus*	*Rhizophora apiculata*	Rhizophoraceae	Thailand	[73]
*Fissuroma aggregatum*	*Phyllostachys reticulata*	Poaceae	Japan	[95,124,125,126,127]
*Fissuroma arengae*	*Arenga pinnata*	Arecaceae	Thailand	[88]
*Fissuroma bambusae*	*Bambusa* spp.	Poaceae	Thailand	[58]
*Fissuroma bambusicola*	*Bambusa* spp.	Poaceae	China	[128]
*Fissuroma calami*	*Calamus rotang*	Arecaceae	Thailand	[59]
*Fissuroma caryotae*	*Arenga undulatifolia Caryota urens*	Arecaceae	China	[59], this study
*Fissuroma chinense*	*Bambusa* spp.	Poaceae	China	[66]
*Fissuroma fissuristomum*	*Bambusa* spp. *Calamus conirostris*, *Licuala* sp., *Mauritia flexuosa*	Arecaceae, Poaceae	Australia, Brunei, China, Ecuador, Papua New Guinea	[73,107,129]
*Fissuroma kavachabeejae*	*Calamus andamanicus*	Arecaceae	India	[105]
*Fissuroma maculans*	*Arenga* spp.	Arecaceae	Philippines	[79,130]
*Fissuroma microsporum*	*Borassus flabellifer*	Arecaceae	India	[105]
*Fissuroma neoaggregatum*	*Bambusa* spp.	Poaceae	Thailand	[58]
*Fissuroma palmae*	*Arenga pinnata*	Arecaceae	Thailand	[86]
*Fissuroma taiwanense*	*Hedychium coronarium*	Zingiberaceae	China	[87])
*Fissuroma thailandicum*	*Bambusa* spp.	Poaceae	Thailand	[58]
*Fissuroma wallichiae*	*Wallichia* sp.	Theaceae	Thailand	[88]
*Neoastrosphaeriella alankrithabeejae*	*Calamus andamanicus*	Arecaceae	India	[105]
*Neoastrosphaeriella aquatica*	*Phoenix paludosa*	Arecaceae	China, Thailand	[89], this study
*Neoastrosphaeriella krabiensis*	*Metroxylon sagu*	Arecaceae	Thailand	[79]
*Neoastrosphaeriella phoenicis*	*Phoenix paludosa*	Arecaceae	Thailand	[86]
*Neoastrosphaeriella sribooniensis*	*Calamus rotang*	Arecaceae	Thailand	[59]
*Posidoniomyces atricolor*	*Posidonia oceanica*	Posidoniaceae	Croatia	[131]
*Rimora mangrovei*	*Avicennia officinalis*, *Rhizophora mangle*	Rhizophoraceae, Verbenaceae	The United States, India	[73,121,122]
*Aquatospora cylindrica*	Unknown host	-	Thailand	[60]
*Astrosphaeriella angustispora*	*Eleiodoxa conferta*, *Licuala* spp.	Arecaceae	Brunei	[119,132,133]
*Astrosphaeriella aosimensis*	*Livistona subglobosa*	Arecaceae	Japan	[134,135]
*Astrosphaeriella applanata*	*Alnus* sp., *Carpinus* sp., *Quercus* spp.	-	Poland, Sweden, the United Kingdom,	[73,109,136]
*Astrosphaeriella aquatica*	*Licuala longicalycata*, *Livistona* spp.	Arecaceae	Ecuador, Papua New Guinea, Thailand	[73,119,133,137]
*Astrosphaeriella asiana*	*Aegiceras corniculatum*, *Sonneratia alba*	Lythraceae, Primulaceae	Thailand	[119,138]
*Astrosphaeriella australiensis*	*Calamus* spp.	Arecaceae	Australia	[119]
*Astrosphaeriella bambusae*	*Bambusa* spp., *Metroxylon sagu*	Arecaceae, Poaceae	China, Thailand	[58], this study
*Astrosphaeriella bambusella*	*Bambusa* spp.	Poaceae	Indonesia	[126,139]
*Astrosphaeriella callicarpa*	Unknown host	-	Indonesia	[140]
*Astrosphaeriella daemonoropis*	*Daemonorops margaritae*	Arecaceae	China	[115,129]
*Astrosphaeriella erumpens*	Unknown host	Arecaceae	Cuba	[141]
*Astrosphaeriella exorrhiza*	*Iriartea* sp.	Arecaceae	Venezuela	[119,142]
*Astrosphaeriella floridana*	*Sabal palmetto*	Arecaceae	The United States, Thailand	[133,143]
*Astrosphaeriella frondicola*	*Calamus* spp., *Daemonorops* spp., *Laccospadix australasica*, *Oraniopsis appendiculata*	Arecaceae	Australia, Brunei, China	[73,115,119,144]
*Astrosphaeriella fuscomaculans*	*Phyllostachys nigra*	Poaceae	Japan	[73,125,126]
*Astrosphaeriella fusispora*	*Bambusa spinosa*, *Phyllostachys bambusoides*, *Pleioblastus pubescens*	Poaceae	Japan, Philippines	[73,125,126,130,145]
*Astrosphaeriella gaofengensis*	*Bambusa* spp.	Poaceae	China	[66]
*Astrosphaeriella immersa*	*Archontophoenix alexandrae*	Arecaceae	China	[115,129]
*Astrosphaeriella lageniformis*	*Cocos nucifera*	Arecaceae	China	[73,146]
*Astrosphaeriella lenticularis*	*Geonoma* sp.	Arecaceae	Brunei, Ecuador	[73,119]
*Astrosphaeriella linguiformis*	*Bambusa* spp.	Poaceae	China	[147]
*Astrosphaeriella livistonicola*	*Livistona chinensis*	Arecaceae	China, Thailand	[73,115,129,133]
*Astrosphaeriella longispora*	Unknown host	-	Costa Rica	[148]
*Astrosphaeriella lophiostomopsis*	*Arenga undulatifolia*	Arecaceae	Brunei, Thailand	[119,133]
*Astrosphaeriella macrospora*	*Miscanthus* spp.	Poaceae	China	[149]
*Astrosphaeriella malayensis*	*Daemonorops* sp., *Licuala longicalycata*	Arecaceae	Malaysia, Papua New Guinea, Thailand	[119,129,133]
*Astrosphaeriella maquilingiana*	*Calamus* spp., *Iriartea* sp.	Arecaceae	Australia, Ecuador, Philippines	[73,119]
*Astrosphaeriella mauritiae*	*Mauritia flexuosa*	Arecaceae	Ecuador	[119]
*Astrosphaeriella minima*	*Bambusa* spp.	Poaceae	China, Indonesia	[140,150]
*Astrosphaeriella minoensis*	*Licuala ramsayi*, *Phyllostachys reticulata*, *Sasa kurilensis*	Arecaceae, Poaceae	Australia, Japan	[119,142,150]
*Astrosphaeriella neofusispora*	*Bambusa* spp.	Poaceae	Thailand	[58]
*Astrosphaeriella neostellata*	*Bambusa* spp.	Poaceae	Thailand	[58]
*Astrosphaeriella nipicola*	*Nipa* sp.	Arecaceae	Indonesia	[119]
*Astrosphaeriella nypae*	*Bambusa* spp., *Nypa fruticans*, *Phoenix hanceana*	Arecaceae, Poaceae	Brunei, China	[73,115,119]
*Astrosphaeriella pallidipolaris*	Unknown host	-	China	[149]
*Astrosphaeriella papuana*	*Bambusa* spp.	Poaceae	Papua New Guinea	[126,150]
*Astrosphaeriella picea*	Unknown host	-	-	[151]
*Astrosphaeriella pinicola*	*Pinus* spp.	Pinaceae	Austria	[152]
*Astrosphaeriella polymorpha*	*Ulmus* spp.	Ulmaceae	The United States	[153]
*Astrosphaeriella roseobrunnea*	*Bambusa* spp.	Poaceae	China	[66]
*Astrosphaeriella seychellensis*	Unknown host	-	Seychelles	[154]
*Astrosphaeriella splendida*	*Arundinaria hindsii*, *Astrocaryum* sp., *Iriartea* sp., *Jessenia bataua*, *Mauritia flexuosa*	Arecaceae, Poaceae	China, Ecuador	[73,107,119]
*Astrosphaeriella stellata*	*Bambusa* spp., *Calamus* spp., *Dendrocalamus* spp., *Phyllostachys heterocycla*, *Thysanolaena maxima*	Arecaceae, Poaceae	Australia, China, India, Japan, Papua New Guinea, Philippines, Vietnam	[107,115,119,127,150,155,156,157]
*Astrosphaeriella striaspora*	*Valota insularis*	Poaceae	Venezuela	[119]
*Astrosphaeriella sundarbanensis*	*Sonneratia apetala*	Lythraceae	India	[73]
*Astrosphaeriella thailandensis*	Unknown host	-	Thailand	[158]
*Astrosphaeriella thailandica*	*Bambusa* spp.	Poaceae	Thailand	[58]
*Astrosphaeriella thysanolaenae*	*Thysanolaena maxima*	Poaceae	Thailand	[58]
*Astrosphaeriella trochus*	*Bambusa* spp., *Phragmites* spp., *Phyllostachys* spp.	Poaceae	China, Indonesia, South Africa	[73,107,119]
*Astrosphaeriella uberina*	Unknown host	Arecaceae	France, Nicaragua	[73,119]
*Astrosphaeriella uniseptata*	*Miliusa tectona*	Annonaceae	India	[159]
*Astrosphaeriella vaginata*	*Bactris baculifera*	Arecaceae	Mexico	[160]
*Astrosphaeriella venezuelensis*	*Bambusa* spp.	Poaceae	Venezuela	[126]
*Astrosphaeriella vesuvius*	*Calamus* spp., *Daemonorops oxycarpus*, *Korthalsia* sp., *Licuala* sp.	Arecaceae	Australia, Brunei, Indonesia, Thailand, Malaysia, Papua New Guinea, Sri Lanka	[73,119]
*Astrosphaeriella yunnanensis*	*Bambusa* spp.	Poaceae	China	[161]
*Astrosphaeriellopsis bakeriana*	*Livistona sinensis*	Arecaceae	Singapore	[162]
*Astrosphaeriellopsis caryotae*	*Caryota* sp.	Arecaceae	Thailand	[59]
*Caryospora aquatica*	Unknown host	-	Thailand	[80]
*Caryospora australiensis*	Unknown host	-	Australia	[163]
*Caryospora coffeae*	*Coffea* sp.	Rubiaceae	Venezuela	[164]
*Caryospora daweiensis*	*Melocalamus compactiflorus*	Poaceae	China	[165]
*Caryospora langloisii*	*Arundinaria* sp., *Grewia asiatica*	Malvaceae, Poaceae	India, The United States	[126,166,167,168]
*Caryospora masonii*	*Eugenia caryophyllus*	Myrtaceae	Tanzania	[169]
*Caryospora minima*	*Amygdalus persica*, *Prunus persica*	Rosaceae	The United States	[168]
*Caryospora obclavata*	Unknown host	-		[104]
*Caryospora phyllostachydis*	*Phyllostachys bambusoides*	Poaceae	Japan	[135]
*Caryospora pruni*	*Prunus persica*	Rosaceae	China	this study
*Caryospora quercus*	*Citus maxima*, *Quercus* sp.	Fagaceae, Rutaceae	China, Thailand	[60,83], this study
*Caryospora submersa*	Unknown host	-	Thailand	[60]
*Javaria samuelsii*	*Nothofagus* sp.	Arecaceae, Nothofagaceae	Brazil, New Zealand	[170,171]
*Javaria shimekii*	Unknown host	-	Nicaragua	[143]
*Mycopepon bambusae*	*Babmbusa* spp.	Poaceae	China	[172]
*Mycopepon fusoidisporus*	*Babmbusa* spp.	Poaceae	China	[172]
*Mycopepon smithii*	Unknown host	-	Nicaragua	[170]
*Pithomyces africanus*	*Borassus* spp., *Ficus* spp., *Hyphaene thebaica*, *Musa balbisiana*, *Ophiopogon japonicus*, *Trachelospermum jasminoides*	Apocynaceae, Arecaceae, Asparagaceae, Moraceae, Musaceae	China, Ghana, Sierra Leone	[73,110,111]
*Pithomyces alabamensis*	*Quercus* spp.	Fagaceae	The United States	[173]
*Pithomyces arecastri*	*Arecastrum romanzoffianum*	Arecaceae	China	[174]
*Pithomyces bulbilus*	*Eucalyptus* spp.	Myrtaceae	India	[175]
*Pithomyces caryotae*	*Caryota* sp.	Arecaceae	Thailand	[59]
*Pithomyces cateniformis*	*Wisteria sinensis*	Fabaceae	China	[110]
*Pithomyces cinnamomeus*	Unknown host	-	Cuba	[176]
*Pithomyces clavisporopsis*	*Lilium amoenum*	Liliaceae	China	[110]
*Pithomyces clavisporus*	Unknown host	-	The United States	[177]
*Pithomyces cupaniae*	*Actinodaphne angustifolia*, *Albizia ferruginea*, *Anisophyllea laurina*, *Carpodinus hirsuta*, *Clitandra* sp., *Cupania guatemalensis*, *Funtumia africana*, *Jasminum dichotomum*, *Millettia pallens*, *Sorindeia juglandifolia*	Anacardiaceae, Anisophylleaceae, Apocynaceae, Fabaceae, Lauraceae, Oleaceae, Sapindaceae	Costa Rica, Myanmar, Sierra Leone	[73,111,178]
*Pithomyces dimorphosporus*	Unknown host	-	Brazil	[179]
*Pithomyces divaricatus*	*Casearia tomentosa*	Salicaceae	India	[180]
*Pithomyces djbhatii*	Unknown host	Arecaceae	India	[73]
*Pithomyces elaeidicola*	*Elaeis guineensis*, *Phoenix canariensis*, *Trachycarpus* spp.	Arecaceae	China, Sierra Leone, Tanzania	[73,110,111,181]
*Pithomyces ellipticus*	*Lagerstroemia speciosa*, *Trachycarpus fortunei*	Arecaceae, Lythraceae	China	[73,110]
*Pithomyces ellisii*	*Butea monosperma*, *Eucalyptus* sp.	Fabaceae, Myrtaceae	India	[182]
*Pithomyces flavus*	*Oncosperma* sp.	Arecaceae	Sri Lanka	[73,111]
*Pithomyces gladioli*	*Gladiolus communis*	Iridaceae	China	[73,110]
*Pithomyces graminicola*	*Arachis hypogaea*, *Canna* spp., *Dendrocalamus* sp., *Mangifera indica*, *Panicum* spp., *Persea americana*, *Phyllostachys* spp., *Ravenala* spp., *Saccharum* spp., *Sporobolus fertilis*	Anacardiaceae, Cannaceae, Fabaceae, Lauraceae, Poaceae, Strelitziaceae	China, Fiji, India, South Africa	[73,110,111,112,113,114,115]
*Pithomyces helminthosporioides*	*Ficus elastica*	Moraceae	China	[73,110]
*Pithomyces hyalosporus*	Unknown host	-	India	[159]
*Pithomyces leprosus*	*Faurea saligna*	Proteaceae	Tanzania	[183]
*Pithomyces licualae*	*Licuala* sp.	Arecaceae	China	[59]
*Pithomyces longiclavisporus*	Soil	-	China	[117]
*Pithomyces longipes*	*Bambusa ventricosa*	Poaceae	China	[174]
*Pithomyces musae*	*Musa wilsonii*	Musaceae	China	[73]
*Pithomyces niger*	*Bambusa* spp.	Poaceae	Cuba	[184]
*Pithomyces obpyriformis*	*Lagerstroemia speciosa*	Lythraceae	China	[174]
*Pithomyces obscuriseptatus*	*Acorus calamus*, *Butomus umbellatus*, *Carex* spp., *Cyperus fuscus*, *Eleocharis* spp., *Juncus* spp., *Sparganium* spp.	Acoraceae, Arecaceae, Butomaceae, Cyperaceae, Juncaceae, Typhaceae	Peru, Poland	[73,116]
*Pithomyces pallidus*	Soil	-	China	[117]
*Pithomyces prolatus*	*Pithecellobium cubense*	Fabaceae	Cuba	[185]
*Pithomyces pulvinatus*	*Ficus microcarpa*, *Phoenix* sp., *Setaria pumila*	Arecaceae, Fabaceae, Moraceae, Poaceae	China, Indonesia, Pakistan, Poland	[73,109,110,186]
*Pithomyces quadratus*	*Crataegus* sp.	Rosaceae	The United States	[187]
*Pithomyces saccharicola*	*Saccharum officinarum*	Poaceae	China	[174]
*Pithomyces sivaramaprasadii*	Unknown host	-	India	[188]
*Pithomyces subramanianii*	Unknown host	-	India	[73]
*Pithomyces sumiderensis*	Unknown host	-	Cuba	[176]
*Pithomyces taiwanensis*	*Arecastrum romanzoffianum*	Arecaceae	China	[73,110,189]
*Pithomyces trachelospermi*	*Trachelospermum jasminoides*	Apocynaceae	China	[174]
*Pithomyces valparadisiacus*	*Calopsis* spp., *Leucadendron* sp., *Puya chilensis*, *Puya coerulea*, *Rhodocoma capensis*	Bromeliaceae, Proteaceae, Restionaceae	Chile, South Africa	[73,190,191]
*Pithomyces variegatae*	*Bauhinia variegata*	Fabaceae	China	[174]
*Pteridiospora bambusae*	*Bambusa* sp.	Poaceae	China	[90]
*Pteridiospora chiangraiensis*	*Bambusa* sp.	Poaceae	Thailand	[58]
*Pteridiospora chochrjakovii*	*Quercus pedunculiflora*	Fagaceae	Azerbaijan	[73]
*Pteridiospora javanica*	*Bambusa* sp.	Poaceae	Indonesia, Thailand	[91,126]
*Pteridiospora munkii*	*Phoenix sylvestris*	Arecaceae	India	[121]
*Pteridiospora spinosispora*	*Fraxinus pennsylvanica*, *Liquidambar styraciflua*	Altingiaceae, Oleaceae	The United States	[192]
*Quercicola fusiformis*	*Quercus* sp.	Fagaceae	Thailand	[83]
*Quercicola guttulospora*	-	Fagaceae	Thailand	[83]
*Xenoastrosphaeriella aquatica*	Unknown host	-	China	[193]
*Xenoastrosphaeriella tornata*	*Bambusa* sp.	Poaceae	Thailand	[58,194]
*Carinispora nypae*	*Licuala longicalycata*, *Nypa fruticans*	Arecaceae	Brunei, Thailand	[97,133,195]
*Carinispora velatispora*	*Oncosperma tigillarium*	Arecaceae	Brunei	[137]
*Pseudoastrosphaeriella aequatoriensis*	*Phytelephas* sp.	Arecaceae	Ecuador	[73,119]
*Pseudoastrosphaeriella africana*	*Arenga undulatifolia*, *Calamus* spp., *Daemonorops* sp., *Phragmites australis*	Arecaceae, Poaceae	Australia, Brunei, Malaysia, Philippines, Tanzania	[119,142]
*Pseudoastrosphaeriella aquatica*	Unknown host	-	Thailand	[60]
*Pseudoastrosphaeriella bambusae*	*Bambusa* spp.	Poaceae	Thailand	[58]
*Pseudoastrosphaeriella longicolla*	*Bambusa* spp.	Poaceae	Thailand	[58]
*Pseudoastrosphaeriella papillata*	*Bambusa* spp.	Poaceae	Brunei	[119]
*Pseudoastrosphaeriella thailandensis*	*Bambusa* spp.	Poaceae	Thailand	[119]
*Pseudoastrosphaeriellopsis kaveriana*	*Avicennia marina*	Arecaceae	India	[58]
*Pseudoastrosphaeriella zingiberacearum*	*Hedychium coronarium*	Zingiberaceae	China	this study

## 4. Discussion

The families Aigialaceae, Astrosphaeriellaceae, and Pseudoastrosphaeriellaceae are notable within the order Pleosporales, as they all possess carbonaceous ascomata and trabeculate pseudoparaphyses [58,93,196]. Of them, Aigialaceae was established by Suetrong et al. [75] to include three genera, *Aigialus*, *Ascocratera*, and *Rimora*, which were collected from mangrove habitats. Liu et al. [79] added *Fissuroma* and *Neoastrosphaeriella* to Aigialaceae from terrestrial habitats. Subsequently, *Posidoniomyces* was accommodated to the family from Mediterranean seagrass [197]. Thus, currently 29 species belonging to six genera are accepted in Aigialaceae [93,98,103]. *Fissuroma* is the most diverse genus in the family with 16 species, and all other genera have fewer than six species (*Aigialus*: 5 species, *Neoastrosphaeriella*: 5 species, *Ascocratera*: 1 species, *Posidoniomyces*: 1 species, *Rimora*: 1 species). Genera within the Aigialaceae family have been observed in diverse environments, including freshwater, mangrove, marine, and terrestrial ecosystems [58,59,60,66]. Aigialaceae species have carbonaceous, conical to hemispherical ascomata with slit-like opening, trabeculate pseudoparaphyses, cylindrical or cylindrical-clavate asci, and hyaline to brown, ellipsoidal to fusiform, septate to muriform ascospores with a sheath or gelatinous appendages around the apical cells [58,75,87,102]. In this study, we have revealed two new host records, *Fissuroma caryotae* and *Neoastrosphaeriella aquatica* from *Hedychium coronarium* (Zingiberaceae) and *Phoenix paludosa* (Arecaceae), respectively.

Phookamsak et al. [58] established Astrosphaeriellaceae to include two genera, such as *Astrosphaeriella* and *Pteridiospora*. The species have carbonaceous, conical ascomata, trabeculate pseudoparaphyses, bitunicate asci, and fusiform or obclavate ascospores. The asexual morph can be either coelomycetous or hyphomycetous [59,93]. The phylogenetic affinity of *Astrosphaeriellopsis* and *Pithomyces* in Astrosphaeriellaceae was discussed by Wanasinghe et al. [59] based on LSU, SSU, and *tef1-α* sequences. In addition, many novel Astrosphaeriellaceae genera were discovered in recent studies. For instance, four genera, such as *Mycopepon* [172], *Quercicola*, *Xenoastrosphaeriella* [83], and *Aquatospora* [60], were established in just three years. The phylogenetic placement of Javaria is still not well-resolved since it is lacking molecular data, but it has been placed in Astrosphaeriellaceae in recent outlines [103]. In addition, the taxonomic placement of the *Caryospora* has been controversial. This was placed in Caryosporaceae based on *C. aquatica* and *C. minima*, but later transferred to Astrosphaeriellaceae due to the similarity of carbonaceous ascostromata and trabeculate pseudoparaphyses [80,83]. The affinity of *Caryospora* to Astrosphaeriellaceae was followed by a recent outline of Wijayawardene et al. [103] as well. Therefore, currently 10 genera are accepted in Astrosphaeriellaceae [60,103]. Of them, some genera are highly diverse (e.g., *Astrosphaeriella*: 55 species, *Pithomyces*: 41 species), while others have a few numbers of species (*Caryospora*: 12 species, *Pteridiospora*: 6 species, *Mycopepon*: 4 species, *Astrosphaeriellopsis*: 2 species, *Javaria*: 2 species, *Quercicola*: 2 species, *Xenoastrosphaeriella*: 2 species, *Aquatospora*: 1 species). In this study, we introduce a new species, *Caryospora pruni*, from the decaying fruit pericarp of *Prunus persica* (Rosaceae), and two new host records, *Astrosphaeriella bambusae* and *Caryospora quercus* from *Hedychium coronarium* (Zingiberaceae) and *Citus maxima* (Rutaceae), respectively.

Pseudoastrosphaeriellaceae consists of three genera, such as *Carinispora*, *Pseudoastrosphaeriella*, and *Pseudoastrosphaeriellopsis*, which have hemispherical to lenticular ascostromata, trabeculate pseudoparaphyses, and brown, fusiform to obclavate ascospores with striations or longitudinal ridges [58,93,118,198]. *Pseudoastrosphaeriella* is the most diverse genus in the family, with seven species, and others have fewer species (*Carinispora*: 2 species and *Pseudoastrosphaeriellopsis*: 1 species). The asexual morph has been recorded as coelomycetous with pycnidial, conical, or hemispherical to globose conidiomata, phialidic, cylindric-clavate or ampulliform conidiogenous cells, and hyaline, globose to subglobose, or oblong, aseptate conidia [58]. The type genus is *Pseudoastrosphaeriella*, which was previously placed in *Astrosphaeriella sensu lato*. In this study, we introduce a new species, *Pseudoastrosphaeriella zingiberacearum*, from the dead stem of *Hedychium coronarium* in China. Interestingly, this is the first Pseudoastrosphaeriellaceae species reported from the host family, Zingiberaceae.

The new species and host records from Guangdong and Jiangxi Provinces extend the known distributions of Aigialaceae, Astrosphaeriellaceae, and Pseudoastrosphaeriellaceae within humid subtropical regions of China. Their occurrence on diverse hosts, Arecaceae, Zingiberaceae, and Rutaceae, suggests these families are not restricted to mangrove or aquatic habitats, but can adapt to terrestrial plant litter environments. This supports the idea that subtropical forests act as biodiversity hotspots for pleosporalean fungi, where microclimatic conditions such as high humidity, seasonal rainfall, and decaying organic matter availability provide suitable niches. Biogeographically, the occurrence of these fungi in subtropical regions of China indicates possible hotspots of fungal diversity within humid subtropical ecosystems. The simultaneous presence of these fungal families in plant litter may result from similar ecological preferences, such as high humidity, nutrient-rich decaying substrates, and stable microhabitats, which together facilitate their coexistence.

Future studies with broader geographic sampling, the use of multi-gene (e.g., ITS, *rpb2*), environmental, or metabarcoding analyses would improve the understanding of fungal diversity and ecological interactions of these families. Since the host specificity of these proposed taxa remains unconfirmed, it could be validated through expanded sampling. Incorporating metagenomics and high-throughput sequencing would also allow for the detection of non-cultivable species, enabling a comprehensive assessment of fungal communities in plant litter and other substrates.

## 5. Conclusions

The morphological characteristics of the new species (*Caryospora pruni* and *Pseudoastrosphaeriella zingiberacearum*), which are introduced in this study, tally well with their respective generic concepts and can be distinguished from related species in their morphology and DNA molecular data. The new host records (*Astrosphaeriella bambusae*, *Caryospora quercus*, *Fissuroma caryotae*, and *Neoastrosphaeriella aquatica*) also provide similar morphological characteristics to their type species, and multi-gene phylogeny analyses also offer evidence for their placements. These discoveries significantly expand our understanding of fungal biodiversity across diverse hosts, substrates, and habitats, which is crucial for ecological research and conservation efforts. Future collections are required to understand the circumscription of some Aigialaceae, Astrosphaeriellaceae, and Pseudoastrosphaeriellaceae genera, which have single species, such as *Ascocratera*, *Aquatospora*, *Posidoniomyces*, *Pseudoastrosphaeriellopsis*, and *Rimora*. Although some genera are highly diverse with numerous species, most of them lack molecular data. For instance, *Pithomyces* comprises 41 species, but molecular data are available for only 7 of them. Similarly, *Astrosphaeriella* includes 55 species, with molecular data available for only 13 species [93,98]. In addition, some genera (e.g., *Javaria*) are still lacking molecular data, and thus, new collections are needed to clarify their phylogenetic affinities.

## Data Availability

All sequence data are available in NCBI GenBank with the accession numbers given in the manuscript.

## Supplementary Materials

The following supporting information can be downloaded at: https://www.mdpi.com/article/10.3390/jof11120834/s1, File S1: Phylogenetic analyses data.
