# Supplementary material for: Global Diversity, Host Associations, and New Insights into Aigialaceae, Astrosphaeriellaceae, and Pseudoastrosphaeriellaceae
Source: J Fungi (Basel). 2025 Nov 25;11(12):834. doi: 10.3390/jof11120834 (PMC12734075; doi:10.3390/jof11120834)
Supplement: Supplementary file 1 [file jof-11-00834-s001.zip › jof-3981854-supplementary.pdf]

Phylogenetic analyses data

The final ML optimization likelihood value was -17628.163576. There were 30.73% undetermined characters or gaps and 1115 distinct alignment patterns. Estimated base frequencies were A = 0.244847, C = 0.246993, G = 0.281392, T = 0.226768; substitution rates AC = 1.031577, AG = 3.099502, AT = 1.059889, CG = 1.274711, CT = 9.314116, GT = 1.000; proportion of invariable sites I = 0.462045; gamma distribution shape parameter α = 0.545508.
